# The risk and protective factors on the mental health of healthcare workers during the lockdown period due to covid-19 pandemic

**DOI:** 10.1038/s41598-024-62288-5

**Published:** 2024-05-21

**Authors:** Kaiwen Liang, Yahui Yang, Kaichao Chen, Furong Lv, Lian Du

**Affiliations:** 1 Health Management Center, The First Affiliated Hospital of Chongqing Medical University, Chongqing, 400016 China; 2 Department of Psychiatry, The First Affiliated Hospital of Chongqing Medical University, Chongqing, 400016 China; 3 Department of Radiology, The First Affiliated Hospital of Chongqing Medical University, Chongqing, 400016 China

**Keywords:** Mental health, Healthcare workers, Adult attachment style, Social support, Health occupations, Risk factors

## Abstract

This study aimed to analyze the impact of the lockdown period due to COVID-19 pandemic on the mental health status of healthcare workers and identify the related risk factors of psychosomatic distress. We conducted an online questionnaire survey to investigate the general demographic characteristics, perceived stress level, adult attachment style (AAS), family cohesion and adaptability, social support, sleep state, emotional state, and physical health of healthcare workers during the lockdown period due to the pandemic in 2022. We compared the mental health status between doctors and nurses, and further analyzed the factors influencing sleep, emotions, physical symptoms, and severe psychosomatic distress separately. For factors that showed statistical significance in the univariate analysis, forward stepwise regression was used for logistic regression analysis to identify risk factors for the corresponding issues. A total of 622 healthcare workers participated in the survey. Among the participants, 121 (19.5%) reported sleep problems, 209 (33.6%) had negative emotional states, and 147 (23.6%) reported physical health problems. There were 48 (7.7%) healthcare workers with severe psychosomatic distress. Compared to the group of nurses, the group of doctors exhibit a higher prevalence of emotional issues, physical health problems and psychosomatic distress. Perceived stress was identified as a risk factor for sleep disturbance, while living with others during quarantine and family adaptability were identified as protective factors. Higher educational background and perceived stress were identified as risk factors for negative emotion, while subjective support was identified as a protective factor. Perceived stress and coming from a rural area were also identified as risk factors for physical health. Overall, for the comparison between the no psychosomatic distress and severe psychosomatic distress groups, perceived stress was identified as a risk factor for severe psychosomatic distress, while subjective support was identified as a protective factor. Healthcare workers’ potential mental and physical health problems are related to their educational background, family cohesion and adaptability, perceived stress and social support. This makes it clearer on how to deal with and prevent adverse consequences when facing stressful situations.

## Introduction

Professions in the medical industry are commonly recognized as high-pressure positions, bringing significant occupational stress to healthcare workers. Healthcare workers, who work in high-pressure environments for long periods, endure immense work-related and mental stress, making them more susceptible to psychological issues^1–3^.Especially with the sudden outbreak of the COVID-19 pandemic, healthcare workers have become one of the highest-risk groups for infection, greatly impacting their mental health and normal work^4^.

Furthermore, a large body of research indicates that the mental health of healthcare workers deteriorated during the pandemic and periods of isolation^5–9^. However, the mental health outcomes for individuals following exposure to stress can vary significantly. Not everyone exposed to similar stressful life events will experience negative outcomes or a decline in functioning^10,11^. Therefore, it is necessary to understand the factors related to the physical and mental issues of healthcare workers when facing stress.

Attachment theory suggests that the intimacy patterns established with caregivers in early infancy is crucial for social and emotional development, providing a template for continuing emotional, cognitive, and behavioral patterns in adulthood, known as adult attachment styles (AASs)^12^. The family environment^15^ and perceived pressure also have a significant impact on an individual’s psychological well-being when facing stress. Our recent research has found that adult attachment styles can directly and indirectly influence the psychological well-being of healthcare workers facing epidemic lockdowns^13^. However, the impact of perceived stress and family environment on their response to such stress is still unclear. Furthermore, how attachment styles, perceived stress, family environment, and other factors interact to influence an individual’s response to stress remains uncertain.

Therefore, this study aimed to explore factors that may affect the mental health of healthcare workers during the lockdown period due to COVID-19 pandemic, which may give us some hints on how to deal with stress and possibly prevent the occurrence of mental and physical problems under stress.

## Results

### Demography characteristics and mental health state of participants

A total of 625 doctors and nurses completed the survey, and 3 subjects were excluded due to irregular questionnaire completion and obvious information errors, such as questions about age being answered with a name. Thus, 622 participants [doctors (n = 378) and nurses (n = 244)] were included in the data analysis, with a questionnaire effective rate of 99.5%. The participants’ mean age was 38.97 years (SD 10.028; range 18–75), of whom 174 (28%) were men and 448 (72%) were women. The majority of participants had a bachelor’s degree or higher (84.7%), and the primary family type was nuclear families (93.2%), defined as a living situation involving both parents rather than parental separation or a single parent (Table 1). Among the 622 participants, 121 (19.5%) reported having sleep problems, 209 (33.6%) had negative emotional states, and 147 (23.6%) self-reported physical health problems (Table 2). There were 48 (7.7%) participants with severe general psychosomatic distress (Table 3).

**Table 1:** Sociodemographic and psychometric characteristics between doctors and nurses.

Variables	Total (N = 622)	Vocation		t/${\chi }^{2}$/Z	*p* value
		doctor [n = 378 (60.8%)]	Nurse [n = 244 (39.2%)]		
Gender				133.931	< 0.001 *
Female	448 (72.0%)	209 (55.3%)	239 (98.0%)		
Male	174 (28.0%)	169 (44.7%)	5 (2.0%)		
Age	38.97 ± 10.028	40.75 ± 10.431	36.21 ± 8.698	5.862	< 0.001 *
Permanent address				18.628	< 0.001 *
Rural	103 (16.6%)	50 (13.2%)	53 (21.7%)		
County	138 (22.2%)	71 (18.8%)	67 (27.5%)		
Urban	381 (61.3%)	257 (68.0%)	124 (50.8%)		
The only child				0.753	0.386
No	448 (72.0%)	277 (73.3%)	171 (70.1%)		
Yes	174 (28.0%)	101 (26.7%)	73 (29.9%)		
Educational background				− 13.159	< 0.001 *
Else	10 (1.6%)	8 (2.1%)	2 (0.8%)		
Junior college	85 (13.7%)	26 (6.9%)	59 (24.2%)		
Bachelor’s degree	293 (47.1%)	118 (31.2%)	175 (71.7%)		
Master’s degree	103 (16.6%)	95 (25.1%)	8 (3.3%)		
Doctor’s degree	131 (21.1%)	131 (21.1%)	0 (0.0%)		
Primary family				1.421	0.491
Nuclear family	580 (93.2%)	356 (94.2%)	224 (91.8%)		
Blended family	22 (3.5%)	12 (3.2%)	10 (4.1%)		
Single-parent family	20 (3.2%)	10 (2.6%)	10 (4.1%)		
Household income level				− 1.589	0.112
Not good	30 (4.8%)	23 (6.1%)	7 (2.9%)		
Not very good	34 (5.5%)	20 (5.3%)	14 (5.7%)		
Average	462 (74.3%)	282 (74.6%)	180 (73.8%)		
Good	85 (13.7%)	47 (12.4%)	38 (15.6%)		
Very good	11 (1.8%)	6 (1.6%)	5 (2.0%)		
Living with parents during 0–3 year-old				1.435	0.231
No	101 (16.2%)	56 (14.8%)	45 (18.4%)		
Yes	521 (83.8%)	322 (85.2%)	199 (81.6%)		
Living with others during quarantine				1.862	0.172
No	86 (13.8%)	58 (15.3%)	28 (11.5%)		
Yes	536 (86.2%)	320 (84.7%)	216 (88.5%)		
Adult attachment style				0.367	0.545
Secure	482 (78.4%)	296 (78.3%)	186 (76.2%)		
Insecure	140 (21.6%)	82 (21.7%)	58 (23.8%)		
Social support rate scale					
Objective support	11.25 ± 4.164	11.16 ± 3.601	11.38 ± 4.916	- 0.642	0.521
Subjective support	24.00 ± 4.649	23.97 $\pm$ 4.437	24.06 $\pm$ 4.969	- 0.228	0.820
Utilization of support	7.78 $\pm$ 1.871	7.67 $\pm$ 1.871	7.95 $\pm$ 1.864	- 1.791	0.074
Family cohesion	71.13 $\pm$ 11.164	70.98 $\pm$ 11.041	71.37 $\pm$ 11.371	- 0.428	0.669
Family adaptability	50.95 $\pm$ 9.799	50.59 $\pm$ 9.663	51.50 $\pm$ 9.999	- 1.136	0.256
Perceived stress					
Perceived stress scores	5.38 ± 2.533	5.54 $\pm$ 2.601	5.15 $\pm$ 2.411	1.876	0.061
Perceived stress—threshold 8				2.513	0.113
Normal	487 (78.3%)	288 (76.2%)	199 (81.6%)		
Severe stress	135 (21.7%)	90 (23.8%)	45 (18.4%)		
Sleep state				3.085	0.079
Normal	501 (80.5%)	296 (78.3%)	205 (84.0%)		
Abnormal	121 (19.5%)	82 (21.7%)	39 (16.0%)		
Emotional state				13.314	< 0.001 *
Normal	413 (66.4%)	230 (60.8%)	183 (75.0%)		
Abnormal	209 (33.6%)	148 (39.2%)	61 (25.0%)		
Physical health state				8.037	0.005 *
Normal	475 (76.4%)	274 (72.5%)	201 (82.4%)		
Abnormal	147 (23.6%)	104 (27.5%)	43 (17.6%)		
Psychosomatic distress				16.109	< 0.001 *
No	336 (54.0%)	182 (48.1%)	154 (63.1%)		
Moderate	238 (38.3%)	158 (41.8%)	80 (32.8%)		
Severe	48 (7.7%)	38 (10.1%)	10 (4.1%)		

*Represents statistical significance.

**Table 2:** Univariate analysis of mental health in healthcare workers.

Variables	Sleep state		*P* value	Emotional state		*P* value	Physical health state		*P* value
	Normal (n = 501)	Abnormal (n = 121)		Normal (n = 413)	Abnormal (n = 209)		Normal (n = 475)	Abnormal (n = 147)	
Sex			0.077			0.002*			0.415
Female	353 (70.5%)	95 (78.5%)		314 (76.0%)	134 (64.1%)		346 (72.8%)	102 (69.4%)	
Male	148 (29.5%)	26 (21.5%)		99 (24.0%)	75 (35.9%)		129 (27.2%)	45 (30.6%)	
Age	38.99 $\pm$ 10.048	38.89 $\pm$ 9.986	0.927	39.20 $\pm$ 10.277`	38.50 $\pm$ 9.524	0.399	39.14 $\pm$ 10.261	38.40 $\pm$ 9.247	0.409
Permanent address			0.707			0.034*			0.003*
Rural	84 (16.8%)	19 (15.7%)		61 (14.8%)	42 (20.1%)		70 (14.7%)	33 (22.4%)	
County	114 (22.8%)	24 (19.8%)		103 (24.9%)	35 (16.7%)		119 (25.1%)	19 (12.9%)	
Urban	303 (69.5%)	78 (64.5%)		249 (60.3%)	132 (63.2%)		286 (60.2%)	95 (64.6%)	
The only child			0.477			0.920			0.655
No	364 (72.7%)	84 (69.4%)		298 (72.2%)	150 (71.8%)		340 (71.6%)	108 (73.5%)	
Yes	137 (27.3%)	37 (30.6%)		115 (27.8%)	59 (28.2%)		135 (28.4%)	39 (26.5%)	
Educational background			0.395			$<$ 0.001*			0.003*
Else	9 (1.8%)	1 (0.8%)		8 (1.9%)	2 (1.0%)		8 (1.7%)	2 (1.4%)	
Junior college	71 (14.2%)	14 (11.6%)		67 (16.2%)	18 (8.6%)		71 (14.9%)	14 (9.5%)	
Bachelor’s degree	237 (47.3%)	56 (46.3%)		205 (49.6%)	88 (42.1%)		234 (49.3%)	59 (40.1%)	
Master’s degree	77 (15.4%)	26 (21.5%)		55 (13.3%)	48 (23.0%)		69 (14.5%)	34 (23.1%)	
Doctor’s degree	107 (21.4%)	24 (19.8%)		78 (18.9%)	53 (25.4%)		93 (19.6%)	38 (25.9%)	
Primary family			0.257			0.086			0.548
Nuclear family	467 (93.2%)	113 (93.4%)		391 (94.7%)	189 (90.4%)		441 (92.8%)	139 (94.6%)	
Blended family	20 (4.0%)	2 (1.7%)		13 (3.1%)	9 (4.3%)		19 (4.0%)	3 (2.0%)	
Single-parent family	14 (2.8%)	6 (5.0%)		9 (2.2%)	11 (5.3%)		15 (3.2%)	5 (3.4%)	
Household income level			0.067			0.001*			0.023*
Not good	20 (4.0%)	10 (8.3%)		17 (4.1%)	13 (6.2%)		22 (4.6%)	8 (5.4%)	
Not very good	23 (4.6%)	11 (9.1%)		15 (3.6%)	19 (9.1%)		20 (4.2%)	14 (9.5%)	
Average	380 (75.8%)	82 (67.8%)		307 (74.3%)	155 (74.2%)		354 (74.5%)	108 (73.5%)	
Good	68 (13.6%)	17 (14.0%)		64 (15.5%)	21 (10.0%)		70 (14.7%)	15 (10.2%)	
Very good	10 (2.0%)	1 (0.8%)		10 (2.4%)	1 (0.5%)		9 (1.9%)	2 (1.4%)	
Living with parents during 0–3 year-old			0.232			0.635			0.117
No	77 (15.4%)	24 (19.8%)		65 (15.7%)	36 (17.2%)		71 (14.9%)	30 (20.4%)	
Yes	424 (84.6%)	97 (80.2%)		348 (84.3%)	173 (82.8%)		404 (85.1%)	117 (79.6%)	
Living with others during quarantine			0.007*			0.046*			0.201
No	60 (12.0%)	26 (21.5%)		49 (11.9%)	37 (17.7%)		61 (12.8%)	25 (17.0%)	
Yes	441 (88.0%)	95 (78.5%)		364 (88.1%)	172 (82.3%)		414 (87.2%)	122 (83.0%)	
Adult attachment style			$<$ 0.001*			0.015*			0.014*
Secure	403 (80.4%)	79 (65.3%)		332 (80.4%)	150 (71.8%)		379 (79.8%)	103 (70.1%)	
Insecure	98 (19.6%)	42 (34.7%)		81 (19.6%)	59 (28.2%)		96 (20.2%)	44 (29.9%)	
Social support rate scale									
Objective support	11.42 $\pm$ 4.249	10.53 $\pm$ 3.724	0.034*	11.24 $\pm$ 3.506	11.26 $\pm$ 5.724	0.947	11.19 $\pm$ 3.537	11.44 $\pm$ 5.752	0.532
Subjective support	24.34 $\pm$ 4.561	22.62 $\pm$ 4.775	$<$ 0.001*	24.57 $\pm$ 4.662	22.88 $\pm$ 4.425	$<$ 0.001*	24.29 $\pm$ 4.737	23.09 $\pm$ 4.243	0.006*
Utilization of support	7.88 $\pm$ 1.855	7.35 $\pm$ 1.883	0.005*	7.98 $\pm$ 1.871	7.39 $\pm$ 1.816	$<$ 0.001*	7.85 $\pm$ 1.884	7.54 $\pm$ 1.814	0.072
Family cohesion	71.96 $\pm$ 10.750	67.68 $\pm$ 12.190	$<$ 0.001*	72.48 $\pm$ 11.076	68.45 $\pm$ 10.876	$<$ 0.001*	71.59 $\pm$ 11.266	69.63 $\pm$ 10.730	0.063
Family adaptability	51.71 $\pm$ 9.446	47.78 $\pm$ 10.605	$<$ 0.001*	52.01 $\pm$ 9.644	48.85 $\pm$ 9.786	$<$ 0.001*	51.28 $\pm$ 9.971	49.88 $\pm$ 9.172	0.132
Perceived stress scores	5.08 $\pm$ 2.458	6.65 $\pm$ 2.455	$<$ 0.001*	4.35 $\pm$ 2.261	7.42 $\pm$ 1.660	$<$ 0.001*	4.79 $\pm$ 2.437	7.30 $\pm$ 1.784	$<$ 0.001*
Perceived stress—Threshold 8			$<$ 0.001*			$<$ 0.001*			$<$ 0.001*
Normal	415 (82.8%)	72 (59.5%)		377 (91.3%)	110 (52.6%)		410 (86.3%)	77 (52.4%)	
Severe stress	86 (17.2%)	49 (40.5%)		36 (8.7%)	99 (47.4%)		65 (13.7%)	70 (47.6%)	

*Represents statistical significance.

**Table 3:** Univariate analysis of the general psychosomatic states of healthcare workers.

Variables	Total	No psychosomatic distress	Moderate psychosomatic distress	Severe psychosomatic distress	F/${\chi }^{2}$/H	*P* value
Overall	622	336 (54.0%)	238 (38.3%)	48 (7.7%)		
Sex					3.670	0.160
Female	448 (72.0%)	251 (74.7%)	161 (67.6%)	36 (75.0%)		
Male	174 (28.0%)	85 (25.3%)	77 (32.4%)	12 (25.0%)		
Age	38.97 $\pm$ 10.028	39.20 $\pm$ 10.304	38.54 $\pm$ 9.748	39.46 $\pm$ 9.560	0.361	0.697
Permanent address		a			10.794	0.029*
Rural	103 (16.6%)	47 (14.0%)	50 (21.0%)	6 (12.5%)		
County	138 (22.2%)	88 (26.2%)	42 (17.6%)	8 (16.7%)		
Urban	381 (61.3%)	201 (59.8%)	146 (61.3%)	34 (70.8%)		
The only child					0.023	0.989
No	448 (72.0%)	242 (72.0%)	171 (71.8%)	35 (72.9%)		
Yes	174 (28.0%)	94 (28.0%)	67 (28.2%)	13 (27.1%)		
Educational background		a, b			12.934	0.002*
Else	10 (1.6%)	7 (2.1%)	3 (1.3%)	0 (0.0%)		
Junior college	85 (13.7%)	54 (16.1%)	28 (11.8%)	3 (6.3%)		
Bachelor’s degree	293 (47.1%)	171 (50.9%)	101 (42.4%)	21 (43.8%)		
Master’s degree	103 (16.6%)	41 (12.2%)	50 (21.0%)	12 (25.0%)		
Doctor’s degree	131 (21.1%)	63 (18.8%)	56 (23.5%)	12 (25.4%)		
Primary family					7.050	0.106
Nuclear family	580 (93.2%)	319 (94.9%)	214 (89.9%)	47 (97.9%)		
Blended family	22 (3.5%)	10 (3.0%)	12 (5.0%)	0 (0.0%)		
Single-parent family	20 (3.2%)	7 (2.1%)	12 (5.0%)	1 (2.1%)		
Household income level		a			10.489	0.005*
Not good	30 (4.8%)	14 (4.2%)	13 (5.5%)	3 (6.3%)		
Not very good	34 (5.5%)	10 (3.0%)	17 (7.1%)	7 (14.6%)		
Average	462 (74.4%)	251 (74.7%)	179 (75.2%)	32 (66.7%)		
Good	85 (13.7%)	52 (15.5%)	28 (11.8%)	5 (10.4%)		
Very good	11 (1.8%)	9 (2.7%)	1 (0.4%)	1 (2.1%)		
Living with parents during 0–3 year-old					2.086	0.352
No	101 (16.2%)	50 (14.9%)	40 (16.8%)	11 (22.9%)		
Yes	521 (83.8%)	286 (85.1%)	198 (83.2%)	37 (77.1%)		
Living with others during quarantine		b	b		10.551	0.005*
No	86 (13.8%)	40 (11.9%)	32 (13.4%)	14 (29.2%)		
Yes	536 (86.2%)	296 (88.1%)	206 (86.6%)	34 (70.8%)		
Adult attachment style		b	b		13.105	0.001*
Secure	482 (77.5%)	273 (81.3%)	181 (76.1%)	28 (58.3%)		
Insecure	140 (22.5%)	63 (18.8%)	57 (23.9%)	20 (41.7%)		
Social support rate scale						
Objective support	11.25 $\pm$ 4.164	11.29 $\pm$ 3.521	11.42 $\pm$ 4.929	10.10 $\pm$ 4.091	2.030	0.132
Subjective support	24.00 $\pm$ 4.649	24.77 $\pm$ 4.621^a, b^	23.39 $\pm$ 4.555 ^b^	21.65 $\pm$ 4.123	13.361	$<$ 0.001*
Utilization of support	7.78 $\pm$ 1.871	8.02 $\pm$ 1.900^a, b^	7.60 $\pm$ 1.783	7.02 $\pm$ 1.828	7.982	$<$ 0.001*
Family cohesion	71.13 $\pm$ 11.164	72.87 $\pm$ 11.201^a, b^	69.53 $\pm$ 10.340	66.92 $\pm$ 12.667	10.250	$<$ 0.001*
Family adaptability	50.95 $\pm$ 9.799	52.40 $\pm$ 9.585^a, b^	49.46 $\pm$ 9.667	48.17 $\pm$ 10.413	8.556	$<$ 0.001*
Perceived stress						
Perceived stress scores	5.38 $\pm$ 2.533	4.15 $\pm$ 2.250^a, b^	6.53 $\pm$ 1.974^b^	8.31 $\pm$ 1.665	198.495	$<$ 0.001*
Perceived stress—Threshold 8		a, b	b		127.597	$<$ 0.001*
Normal	487 (78.3%)	314 (93.5%)	158 (66.4%)	15 (31.3%)		
Severe stress	135 (21.7%)	22 (6.5%)	80 (33.6%)	33 (68.8%)		

*Represents statistical significance.

^a^Represents statistical difference compared with the moderate psychosomatic distress group, and.

^b^Represents statistical difference compared with the severe psychosomatic distress group.

(after Bonferroni correction for multiple comparisons).

Compared to the doctor group, there was a higher proportion of women in the nurse group (*p* < 0.001); the average age of doctors was older than that of nurses (*p* < 0.001); the number of highly educated doctors was greater than that in the nurse group (*p* < 0.001); and there were also differences in the types of permanent address between doctors and nurses (*p* < 0.001); doctors reported a higher prevalence of emotional problems, physical problems, and general psychosomatic distress compared to nurses (*p* < 0.01). The psychological features (AAS, social support, family cohesion and adaptability, and perceived stress) as well as the sociodemographic characteristics did not show statistical significance between doctors and nurses (*p* > 0.05).

### Univariate analysis of mental health among healthcare workers

#### Sleep state

**Figure 1:**
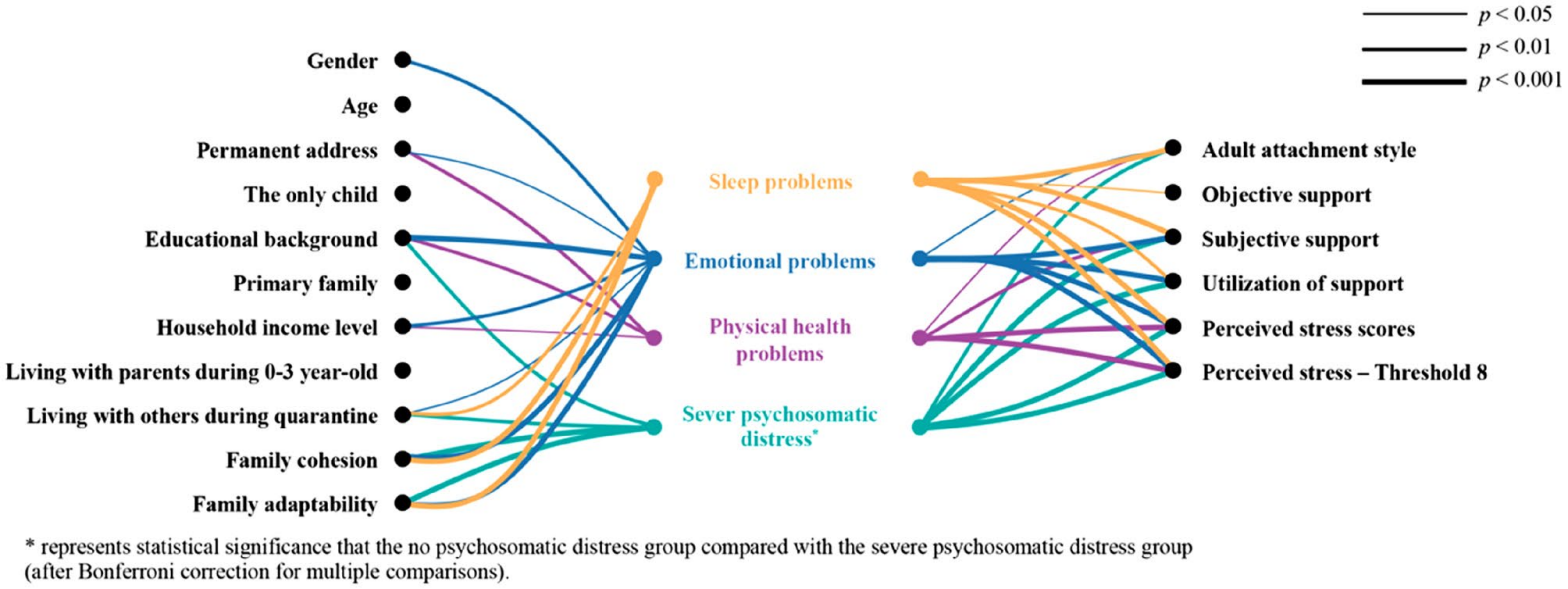
Comparison of significant factors of mental health-related issues among healthcare workers identified in the univariate analysis.

Univariate analysis showed that healthcare workers who lived alone during the lockdown (*p* = 0.007) and with insecure attachment styles (*p* < 0.001) were more likely to have sleep problems. In addition, healthcare workers with sleep problems reported less objective support, subjective support, the utilization of support (*p* < 0.05), and family cohesion and adaptability (*p* < 0.001) than those with normal sleep. Additionally, healthcare workers with sleep problems reported higher perceived stress levels (*p* < 0.001) (Table 2 and Fig. 1).

#### Emotional state

Univariate analysis showed that male healthcare workers were more prone to emotional problems (*p* = 0.002). healthcare workers who lived alone during the lockdown and those with insecure attachment styles were more likely to experience emotional problems (*p* < 0.05). Medical staff with emotional problems reported less subjective support, utilization of support and family cohesion and adaptability (*p* < 0.001) than those with a normal emotional state, rather than objective support (*p* = 0.947). Additionally, healthcare workers with emotional problems reported higher self-perceived pressure (*p* < 0.001). Furthermore, the participants’ permanent addresses, educational backgrounds, and household income levels also had an impact on their emotional states during the epidemic lockdown period (*p* < 0.05) (Table 2 and Fig. 1).

#### Physical health state

Univariate analysis showed that healthcare workers with insecure attachment styles were more likely to experience perceived physical problems (*p* = 0.014). Healthcare workers with physical problems had lower subjective support scores (*p* = 0.006) and more stress (*p* < 0.001) than those with good physical health. Moreover, the participants’ permanent addresses, educational backgrounds, and household income levels also had an impact on their physical health (*p* < 0.05) (Table 2 and Fig. 1).

#### General psychosomatic health state

In our sample, 336 participants (54.0%) were classified into the group of non-psychosomatic distress, while 238 (38.3%) and 48 (7.7%) participants were classified into the moderate and severe psychosomatic distress group, respectively. ANOVAs revealed significant differences among the three groups regarding permanent address (*p* = 0.029), educational background (*p* = 0.002), household income level (*p* = 0.005), living with others during quarantine (*p* = 0.005), AAS (*p* = 0.001), family cohesion (*p* < 0.001), and family adaptability (*p* < 0.001). In terms of social support, only subjective support (*p* < 0.001) and the utilization of support (*p* < 0.001) were significantly different among the three groups. The three groups also differed in perceived stress (*p* < 0.001) (Table 3 and Fig. 1).

### Multivariable analysis of mental health among healthcare workers

We further conducted multivariate logistic regression analyses, assigning statistically significant factors identified in the univariate analysis as independent variables (Model 1, the specific assignment methods can be found in Supplementary material).

#### Sleep state

Multivariate logistic regression analysis identified perceived stress (OR = 1.293; *p* < 0.001) as a risk factor for sleep problems, whereas living with others during quarantine and family adaptability were protective factors (Table 4).

**Table 4:** Multivariate logistic regression analysis of the mental health state and the psychosomatic health state of healthcare workers.

Variables	Model 1		Model 2	
	OR [95% CI]	*p* value	OR [95% CI]	*p* value
Mental health state				
Sleep state				
Living with others during quarantine	0.586 [0.343 1.001]	0.050	0.566 [0.332 0.966]	0.037
Family adaptability	0.969 [0.949 0.990]	0.003	0.966 [0.947 0.986]	0.001
Perceived stress	1.293 [1.178 1.419]	$<$ 0.001	3.031 [1.954 4.704]	$<$ 0.001
Emotional state				
Educational background	1.400 [1.133 1.729]	0.002	1.491 [1.231 1.807]	$<$ 0.001
Household income level	–	–	0.747 [0.560 0.996]	0.047
Subjective support	0.935 [0.892 0.981]	0.006	0.949 [0.907 0.993]	0.024
Utilization of support	–	–	0.877 [0.783 0.982]	0.023
Perceived stress	2.138 [1.878 2.434]	$<$ 0.001	9.345 [5.926 14.738]	$<$ 0.001
Physical health state				
Permanent address				
Permanent address (1)	1.182 [0.694 2.013]	0.539	1.414 [0.829 2.412]	0.204
Permanent address (2)	0.452 [0.250 0.815]	0.008	0.534 [0.294 0.969]	0.039
Educational background	–	–	1.283 [1.039 1.584]	0.021
Subjective support	–	–	0.950 [0.909 0.993]	0.023
Perceived stress	1.675 [1.504 1.865]	$<$ 0.001	5.397 [3.519 8.278]	< 0.001
General psychosomatic health state				
No psychosomatic distress vs. Moderate psychosomatic distress				
Educational background	1.253 [1.035 1.516]	0.021	1.325 [1.108 1.585]	0.002
Subjective support	0.947 [0.908 0.988]	0.012	–	–
Family cohesion	–	–	0.973 [0.957 0.989]	0.001
Perceived stress	1.678 [1.515 1.859]	$<$ 0.001	7.322 [4.361 12.295]	$<$ 0.001
No psychosomatic distress vs. Severe psychosomatic distress				
Educational background	1.618 [1.064 2.460]	0.025	1.702 [1.136 2.552]	0.010
Subjective support	0.830 [0.751 0.919]	$<$ 0.001	0.823 [0.750 0.902]	$<$ 0.001
Perceived stress	2.897 [2.186 3.839]	$<$ 0.001	36.469 [15.737 84.516]	$<$ 0.001
Moderate psychosomatic distress vs. Severe psychosomatic distress				
Living with others during quarantine	0.349 [0.158 0.773]	0.009	0.366 [0.169 0.791]	0.011
Utilization of support	–	–	0.830 [0.689 0.999]	0.049
Perceived stress	1.769 [1.429 2.190]	$<$ 0.001	4.455 [2.249 8.823]	0.001

Model 1: Perceived stress score represents perceived stress; Model 2: Perceived stress—threshold 8 represents perceived stress.

OR: Odds ratio; CI: Confidence interval.

#### Emotional state

Multivariate logistic regression analysis showed that educational background (OR = 1.400; *p* = 0.002) and perceived stress (OR = 2.138; *p* < 0.001) were risk factors for emotional problems, while subjective support (OR = 0.935; *p* = 0.006) was a protective factor (Table 4).

#### Physical health state

Multivariate logistic regression analysis revealed that perceived stress (OR = 1.675; *p* < 0.001) was identified as a risk factor for physical health problems, and medical staff from urban areas were more likely to experience physical health issues than those from county areas (Table 4).

#### General psychosomatic health state

Multivariate logistic regression analysis identified higher educational background and perceived stress (OR > 1.000, *p* < 0.05) as risk factors for moderate psychosomatic distress compared to no psychosomatic distress, whereas subjective support and family cohesion (OR = 0.947, *p* = 0.012) emerged as protective factors in different models. When comparing the severe psychosomatic distress and no psychosomatic distress group, the results were the same as above. Last, perceived stress (OR = 1.769, *p* < 0.001) was also a risk factor when comparing healthcare workers with severe psychosomatic distress to individuals with moderate distress, whereas living with others during quarantine (OR = 0.349, *p* = 0.009) was a protective factor (Table 4).

### Sensitivity analysis

Based on the results above, it is evident that perceived stress has a discernible impact on each mental health issue. Consequently, we employed the 8 criteria for perceived stress as the evaluation standard for the perceived stress variables in the multivariate analysis (Model 2).

Based on the findings, it is evident that both Model 1 and Model 2 identified the same influencing factors for sleep problems. However, for emotional problems, Model 2 identified additional influencing factors compared to Model 1, specifically family income and utilization of social support. Similarly, for physical health problems, Model 2 identified more influencing factors than Model 1, including educational background and subjective support.

**Figure 2:**
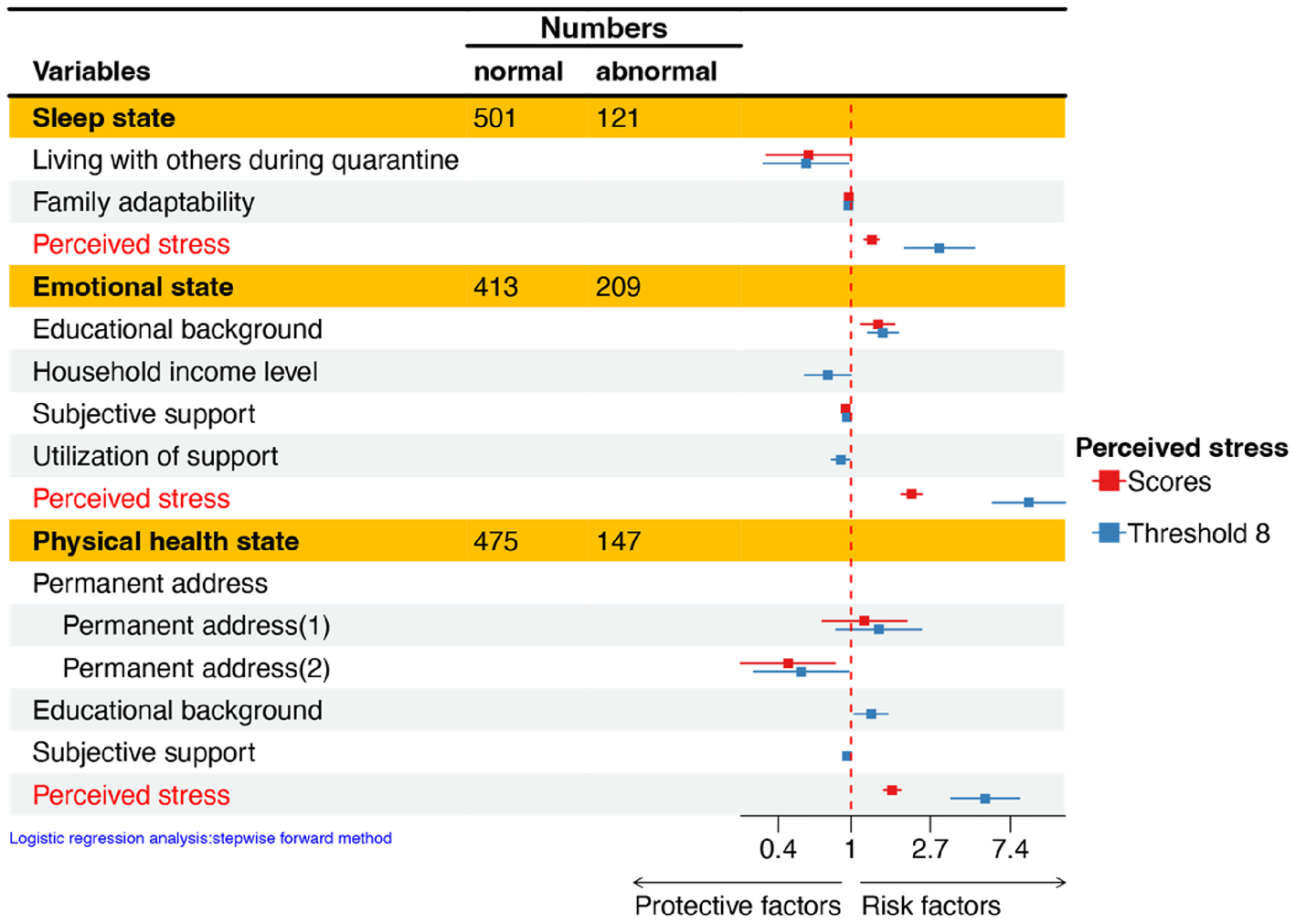
Odds ratios and 95% confidence intervals of the logistic regression analysis of risk and protective factors for mental health problems.

**Figure 3:**
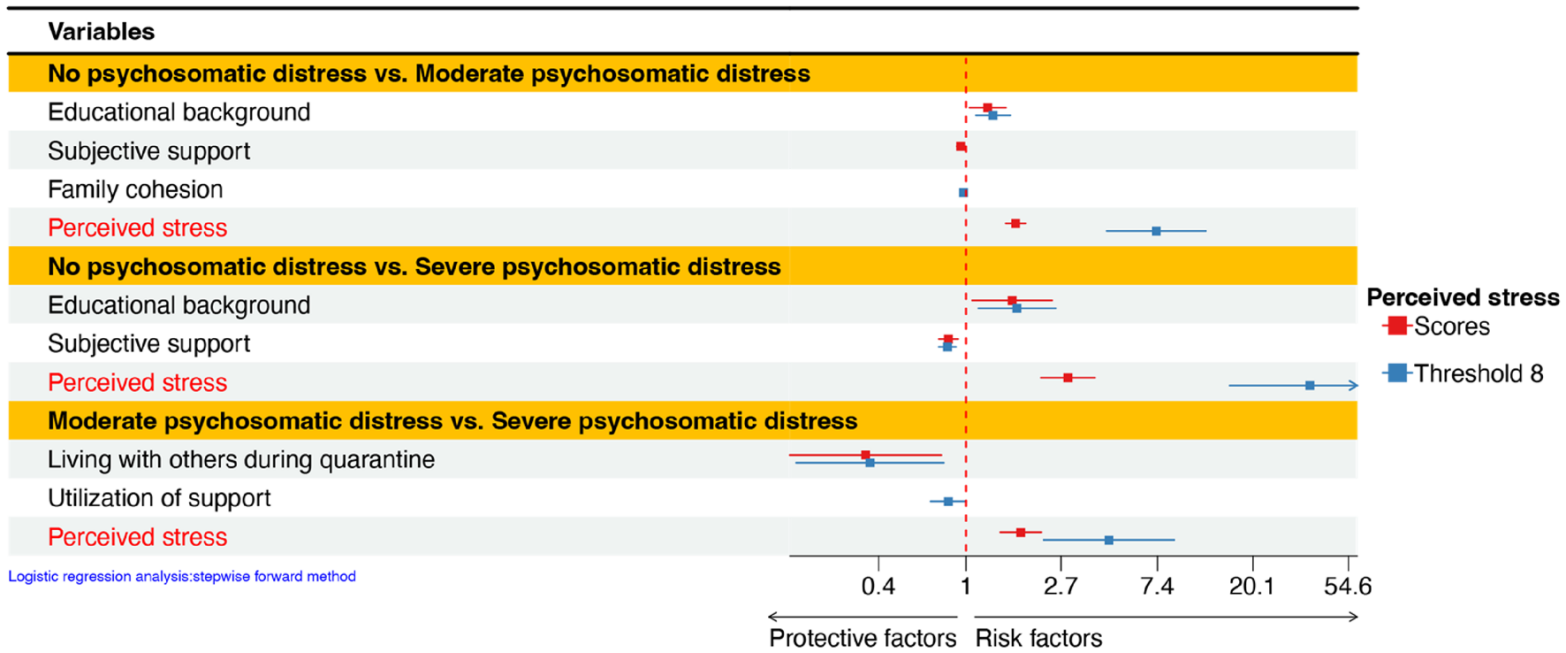
Odds ratios and 95% confidence intervals of the logistic regression analysis of risk and protective factors for psychosomatic distress.

In terms of general psychosomatic health state, when comparing no psychosomatic distress group with moderate psychosomatic distress group, the factors identified by Model 2 differed from those identified by Model 1, added family cohesion and removed subjective support. The Model 2 exhibited similar influencing factors to Model 1 between no psychosomatic distress group and severe psychosomatic distress group, while Model 2 added utilization of social support when compared the moderate psychosomatic distress group to the severe psychosomatic distress group (Table 4, Figs. 2 and 3).

## Discussion

Many studies have focused on the psychological well-being of healthcare workers during the COVID-19 pandemic, often concentrating on specific aspects such as emotions and/or sleep disturbance. However, there is a lack of research at the end of the pandemic. This study was conducted by the end of 2022, and innovatively compared the occurrence of psychological issues from both psychological and physical perspectives between doctors and nurses. Our study indicated that 19.5% of the healthcare workers had sleep problems, 33.6% experienced negative emotional states, 23.6% self-reported physical health issues, and 7.7% suffered from severe general psychosomatic distress. The prevalence of negative emotional of the healthcare workers was similar to previous research, while their sleep problems were relatively mild and their physical discomfort was more severe^14^. This might be related to the different stage of the COVID-19 pandemic, different periods are characterized by unique pressure points. Healthcare workers still worked on the front lines during the period of this research survey, the outlook for the pandemic was relatively clear, and there were sufficient supplies. However, at that time, there were still many uncertainties, medical staff were unable to get sufficient rest and often unable to reunite with family members. This study found doctors reported a higher prevalence of emotional problems, physical problems, and general psychosomatic distressed compared to nurses. This shows a clear difference from the previous meta-analysis^4^. One potential explanation for this is that in this study, the proportion of women in the doctor group was relatively consistent, whereas the nurse group comprised predominantly female nurses with a minimal number of male nurses.

Healthcare workers with a higher education level or higher perceived stress were more likely to experience moderate or severe psychosomatic distress, while family cohesion, subjective support, living with others and higher support utilization were protective factors against moderate and severe psychosomatic distress, respectively. This may be related to the following possibilities. Firstly, healthcare workers with a higher education level may need to engage in solving more complex problems in their professional processes, which could lead to more stress. Secondly, subjective support is a measure of individuals’ subjective evaluations of the support they receive from others, involving their subjective experiences of care, understanding, and acceptance from others; thus, high subjective support scores may be more likely to promote resilience^15,16^. Similarly, living with others during quarantine had similarities with subjective support, as medical staff can support each other. And alleviating the feelings of loneliness also contributes to improved mental health^17^. Thirdly, support utilization measures whether individuals truly seek and use support resources from others when they face difficulties or need help. Lastly, good family relationships also play a positive role in people’s mental health^18^.

Regarding each specific psychological issue, the aforementioned factors continued to act as risk or protective factors. Household income only played a protective role for emotional status under the perceived stress standard threshold of 8; the better an individual’s family economic situation was, the lower their likelihood of developing emotional problems. In terms of physical conditions, healthcare workers from urban areas were less likely to experience physical problems than those from county area. It is worth noting that, among these factors, attachment style did not show any risk or protective effects, even though it showed statistical significance in single-factor analysis across groups, which differs slightly from previous research^10,19^. This may be because attachment often forms in an individual’s early experiences and is influenced by many other social and psychological factors^23–25^.

In addition, there are some issues that may limit the generalizability of our results. First, this was a cross-sectional observational study, lacking longitudinal follow-up. We can only infer the risks and protective factors of mental problems, but cannot confirm their causal relationship. Future longitudinal studies would be valuable in investigating the enduring impact of attachment styles on mental health. Second, the survey relied on online self-report questionnaires. Participants’ responses may be influenced by various factors, such as recall bias. When interpreting the results, it is crucial to take these limitations into account, as self-report measures may not consistently reflect individuals’ true experiences or behaviors^20^. Future research could consider integrating objective measures or utilizing multiple data sources to enhance the validity of the findings. Additionally, the survey only targeted healthcare workers in one large tertiary general hospital. Hence, caution should be exercised when generalizing the results to other populations^21^, and further research is needed to determine its applicability to other populations. Moreover, although all subjects underwent the same quarantine, it is crucial to recognize that the pandemic may have introduced additional stressors, such as a higher incidence of deaths, loss of loved ones, employment in non-professional sectors, and so forth. Future research should aim to integrate this pertinent information to improve the generalizability of the conclusions.

Overall, our survey results indicate that it is possible to predict the potential severity of mental and physical issues among healthcare workers based on their education level, family relationships and social support. This can help in formulating targeted psychological support policies. Additionally, when facing the self-reported perceived stress of healthcare workers at a certain period, it may remind us to give them enough attention and provide appropriate psychological intervention, as perceiving significant pressure can contribute to the development of psychological disturbances.

## Materials and methods

### Participants and procedure

This study, a cross-sectional study, was conducted through an online survey by Questionnaire Star software (https://www.wjx.cn) during the COVID-19 pandemic quarantine in Chongqing. Questionnaire Star software provides an efficient way to collect data from geographically distributed survey participants. Based on previous findings, the first version of the questionnaire was designed after consulting some experts in the field of psychiatry. The questionnaire was then sent to a small group of healthcare workers, and we adjusted several expressions based on their suggestions to form the final version of the questionnaire. Subsequently, from December 12, 2022 to December 30, 2022, the study invitation and data collection forms were connected to a Quick Response code (QR code) distributed across multiple official WeChat work groups of the First Affiliated Hospital of Chongqing Medical University (CMUS), encompassing groups of doctors, nurses, clinical department staff, and medical students. As the subjects of this study were healthcare workers, only questionnaire data from doctors and nurses was included for statistical analysis, with questionnaire data from medical students and administrators omitted from presentation.

Alongside the release of this survey, we emphasized that participation in the survey was voluntary and that individuals could decide whether to fill out the questionnaire. The introduction to the survey was included on the first page of the questionnaire; we also presented the purpose of this study, which was to assess the response of healthcare workers during mandatory isolation following frequent COVID-19 outbreaks, as well as related psychosocial factors. We also introduced the research team and emphasized that the questionnaire survey was anonymous and confidential. At the bottom of the first page, we provided an option for the individual to indicate whether they wanted to participate in the survey or not; only if the participant agreed to participate in the survey could they later complete the questionnaire. Therefore, we did not collect written informed consent, but it can be assumed that the individuals who completed the survey consented to participate voluntarily. To avoid double filling, the questionnaire was also programmed to ensure that participants could only complete the questionnaire once. All methods used in this study were carried out in accordance with relevant guidelines and regulations. And this study was approved by the Ethics Committee of the First Affiliated Hospital of CQMU (K2023-177).

### Instruments

The questionnaire design and survey was supported by the Department of Psychiatry and Health Management Center of the First Affiliated Hospital of CQMU. The questionnaire consisted of two main parts. The first part was a homemade questionnaire that gathered sociodemographic information and assessed the mental health state during the quarantine period. The second part included the following self-assessment scales designed to understand whether AASs and family relationships affect healthcare workers’ responses to stress and what factors contribute to psychosomatic problems.

#### Sleep evaluation

In this study, the sleep state of participants was assessed using three items related to sleep evaluation from the 24-item Hamilton Depression Rating Scale (HAMD-24) ^22^. When a participant’s answer to any item of the 3 items was “lasting more than two weeks” or “nightly”, he or she was considered to have sleep problems.

#### Visual Analogue Scale (VAS)

The visual analogue scale (VAS) is widely used in clinical research to assess and track the subjective experiences of participants (0—no discomfort at all, 10—worst discomfort ever)^23^. VAS have been developed for a broad spectrum of research and clinical purposes, encompassing mood, suicidal intent, depression, anxiety, dyspnea, cigarette cravings, sleep quality, functional capacity, acute pain, chronic pain, nausea, grip strength, disability, and vigor^24,25^. The VAS began to be utilized as a gauge of health-related quality of life starting in the 1970s, subsequent to Priestman and Baum’s^26^ investigation of cancer patients. We used VAS scores to evaluate the participants’ emotional states, physical health, and perceived stress levels. The higher the score was, the worse the participant’s health and the greater their stress. When the score reached 8 or above, it was considered that the participant had relevant health issues.

#### Adult Attachment Scale (AAS)

This study utilized the Revised Adult Attachment Scale (RAAS) developed by Collins in 1996. Previous studies have demonstrated that this scale has good reliability and validity when used in China^27^. The scale comprises 18 items rated on a 5-point scale ranging from 1 (Not at all characteristic of me) to 5 (Extremely characteristic of me), categorized into three subscales: Anxiety, Comfort with closeness, and Comfort with depending on others. According to Brennan et al.^28^, these subscales are further categorized into two dimensions: attachment anxiety and attachment avoidance. Those scoring high on attachment anxiety typically exhibit an exaggerated preoccupation with their own distress and negative emotions, often overreacting to solicit support from others. Conversely, individuals with high attachment avoidance scores tend to adopt a cognitive and behavioral distancing approach from stressful situations, appearing less responsive to them, and eschewing emotional or instrumental support from others^29^. To compare attachment profiles, participants were categorized into their respective attachment styles (secure, preoccupied, dismissing, fearful) depending on whether their scores on the dimensions of attachment-related anxiety and avoidance fell above or below the midpoint of the scale. This study referred to preoccupation patterns, avoidance patterns and fear patterns as insecure attachment patterns, because for each individual, the attachment model was either secure or insecure, and this result was incorporated into the statistical analysis.

#### Social Support Rate Scale (SSRS)

This study assessed participants’ social support using Xiao Shuiyuan’s Social Support Rating Scale (SSRS), which is widely used in China and has a reputation for being reliable and effective^30^. The scale consists of 10 items divided into three dimensions: objective support, subjective support and the utilization of support, with a total score of 0 ~ 66 points; the higher the score is, the higher the social support.

#### The Chinese version of Family Adaptability and Cohesion Scale (FACES II-CV)

The Chinese version of the Family Adaptability and Cohesion Scale, Second Edition (FACES II-CV)^31^ was used in this study to assess participants’ family functioning. This scale evaluates family functioning from two dimensions: family cohesion, that is, the communication, interaction patterns and emotional connections between family members, and family adaptability, that is, the ability of the family system to change accordingly with different family situations and problems encountered in different stages of family development.

#### Perceived stress evaluation

We used VAS scores to evaluate the perceived stress of doctors and nurses. We not only recorded the perceived stress scores but also used a cut-off score of > 8 to distinguish doctors and nurses with severe stress (threshold of 8) according to the VAS criteria^23^.

#### General psychosomatic health evaluation

For evaluating general psychosomatic health, we used the number of coexisting emotional, physical, and sleep problems to determine the severity of a participant’s psychosomatic state. Specifically, (1) participants without any of the above problems were identified as having no psychosomatic distress, (2) participants with 1–2 problems were identified as having moderate psychosomatic distress, and (3) participants with all of the problems were identified as having severe psychosomatic distress.

### Data analysis

In this study, all analyses were conducted using the Statistical Package for Social Sciences (IBM SPSS 26.0). Continuous variables are presented as the mean ± standard deviation ($\overline{x }\pm s$) and were compared using t tests or one-way ANOVA. Categorical variables are presented as frequencies (percentages) [n (%)] and were compared using chi-square tests or Fisher’s exact tests for unordered categorical variables. Ordered categorical variables were compared using the Mann‒Whitney U test or Kruskal‒Wallis H test. Differences were considered statistically significant at *p* < 0.05. We corrected the statistical model for multiple comparisons according to the Bonferroni method (*p* < 0.05/number of comparisons) to minimize the likelihood of type I statistical errors. The significant factors identified in the univariate analysis were further analyzed using a stepwise forward method logistic regression with a removal probability of 0.1 to identify risk factors resulting in sleep problems, emotional issues, physical health problems, and psychosomatic distress, indicated by odds ratios (ORs) and their 95% confidence intervals (CIs).

### Supplementary Information

Supplementary Information.

## Data Availability

The datasets used and/or analyzed during the current study available from the corresponding author on reasonable request.
