# Supplementary material for: The risk and protective factors on the mental health of healthcare workers during the lockdown period due to covid-19 pandemic
Source: Sci Rep. 2024 May 21;14:11628. doi: 10.1038/s41598-024-62288-5 (PMC11109241; doi:10.1038/s41598-024-62288-5)
Supplement: Supplementary file 1 — Supplementary Information. [file 41598_2024_62288_MOESM1_ESM.docx]

**Contents For Supplementary Appendix**

[**Table S1 Assignment method for multivariable logistic regression analysis of mental health-related issues** 3](#_Toc164693471)

[**Table S2 Univariate analysis of sleep state in doctors** 4](#_Toc164693472)

[**Table S3 Univariate analysis of emotional state in doctors** 6](#_Toc164693473)

[**Table S4 Univariate analysis of physical health state in doctors** 8](#_Toc164693474)

[**Table S5 Univariate analysis of psychosomatic state in doctors** 10](#_Toc164693475)

[**Table S6 Multivariate logistic regression analysis of mental health state in doctors** 13](#_Toc164693476)

[**Table S7 Multivariate logistic regression analysis of psychosomatic health state in doctors** 14](#_Toc164693477)

[**Table S8 Univariate analysis of sleep state in nurses** 15](#_Toc164693478)

[**Table S9 Univariate analysis of emotional state in nurses** 17](#_Toc164693479)

[**Table S10 Univariate analysis of physical health state in nurses** 19](#_Toc164693480)

[**Table S11 Univariate analysis of psychosomatic state in nurses** 21](#_Toc164693481)

[**Table S12 Multivariate logistic regression analysis of mental health state in nurses** 24](#_Toc164693482)

[**Table S13 Multivariate logistic regression analysis of psychosomatic health state in nurses** 24](#_Toc164693483)

[**Table S14 Sociodemographic and psychometric characteristics of male healthcare workers** 25](#_Toc164693484)

[**Table S15 Univariate analysis of mental health in male healthcare workers** 27](#_Toc164693485)

[**Table S16 Univariate analysis of psychosomatic state in male healthcare workers** 28](#_Toc164693486)

[**Table S17 Multivariate logistic regression analysis of mental health state in male healthcare workers** 31](#_Toc164693487)

[**Table S18 Multivariate logistic regression analysis of psychosomatic health state in male healthcare workers** 31](#_Toc164693488)

[**Table S19 Sociodemographic and psychometric characteristics of female healthcare workers** 32](#_Toc164693489)

[**Table S20 Univariate analysis of mental health in female healthcare workers** 34](#_Toc164693490)

[**Table S21 Univariate analysis of psychosomatic state in female healthcare workers** 35](#_Toc164693491)

[**Table S22 Multivariate logistic regression analysis of mental health state in female healthcare workers** 38](#_Toc164693492)

[**Table S23 Multivariate logistic regression analysis of psychosomatic health state in female healthcare workers** 38](#_Toc164693493)

[**Figure S1 Comparison of significant factors of mental health-related issues among doctors identified in the univariate analysis** 40](#_Toc164693494)

[**Figure S2 Odds ratios and 95% Confidence Intervals of the logistic regression analysis of risk and protective factors for mental health problem in doctors** 40](#_Toc164693495)

[**Figure S3 Odds ratios and 95% Confidence Intervals of the logistic regression analysis of risk and protective factors for psychosomatic distress in doctors** 41](#_Toc164693496)

[**Figure S4 Comparison of significant factors of mental health-related issues among nurses identified in the univariate analysis** 41](#_Toc164693497)

[**Figure S5 Odds ratios and 95% Confidence Intervals of the logistic regression analysis of risk and protective factors for mental health problem in nurses** 42](#_Toc164693498)

[**Figure S6 Odds ratios and 95% Confidence Intervals of the logistic regression analysis of risk and protective factors for psychosomatic distress in nurses** 42](#_Toc164693499)

[**Figure S7 Comparison of significant factors of mental health-related issues among male doctors and nurses identified in the univariate analysis** 42](#_Toc164693500)

[**Figure S8 Odds ratios and 95% Confidence Intervals of the logistic regression analysis of risk and protective factors for mental health problem in male doctors and nurses** 43](#_Toc164693501)

[**Figure S9 Odds ratios and 95% Confidence Intervals of the logistic regression analysis of risk and protective factors for psychosomatic distress in male doctors and nurses** 43](#_Toc164693502)

[**Figure S10 Comparison of significant factors of mental health-related issues among female doctors and nurses identified in univariate analysis** 43](#_Toc164693503)

[**Figure S11 Odds ratios and 95% Confidence Intervals of the logistic regression analysis of risk and protective factors for mental health problem in female doctors and nurses** 44](#_Toc164693504)

[**Figure S12 Odds ratios and 95% Confidence Intervals of the logistic regression analysis of risk and protective factors for psychosomatic distress in female doctors and nurses** 44](#_Toc164693505)

**Table S1 Assignment method for multivariable logistic regression analysis of mental health-related issues**

| **Variables** | **Assignment method** | |
| --- | --- | --- |
| **Sex** | Female = 0, male = 1 | |
| **Permanent address** | **Dummy variable** | |
|  | **(1)** | **(2)** |
| Rural | 1 | 0 |
| County | 0 | 1 |
| Urban | 0 | 0 |
| **Educational background** | Else = 0, Junior college = 1, Bachelor’s degree = 2,  Master’s degree = 3, Doctor’s degree = 4 | |
| **Household income level** | Not good = 0, Not very good = 1, Average = 2,  Good = 3, Very good = 4 | |
| **Living with others during quarantine** | No = 0, Yes = 1 | |
| **Adult attachment style** | Secure = 0, Insecure = 1 | |
| **Social support rate scale** |  | |
| Objective support | Measured value | |
| Subjective support | Measured value | |
| Utilization of support | Measured value | |
| **Family cohesion** | Measured value | |
| **Family adaptability** | Measured value | |
| **Perceived stress** |  | |
| Perceived stress scores | Measured value | |
| Perceived stress – Threshold 7 | Normal = 0, Severe stress = 1 | |
| **Sleep state** | Normal = 0, Abnormal = 1 | |
| **Emotional state** | Normal = 0, Abnormal = 1 | |
| **Physical health state** | Normal = 0, Abnormal = 1 | |
| **Psychosomatic state** | No psychosomatic distress = 0,  Moderate psychosomatic distress = 1,  Severe psychosomatic distress = 2 | |

**Table S2 Univariate analysis of sleep state in doctors**

| **Variables** | **Total**  **(N = 378)** | **Sleep state** | | **t/**$\boldsymbol{\chi}^{\boldsymbol{2}}$**/Z** | ***p* value** |
| --- | --- | --- | --- | --- | --- |
|  |  | **Normal (n = 296)** | **Abnormal (n = 82)** |  |  |
| **Gender** |  |  |  | 8.567 | 0.003 ^*^ |
| Female | 209 (55.3%) | 152 (51.4%) | 57 (69.5%) |  |  |
| Male | 169 (44.7%) | 144 (48.6%) | 25 (30.5%) |  |  |
| **Age** | 40.75$\pm$10.431 | 41.05$\pm$10.571 | 39.66$\pm$9.896 | 1.067 | 0.287 |
| **Permanent address** |  |  |  | 0.749 | 0.688 |
| Rural | 50 (13.2%) | 37 (12.5%) | 13 (15.9%) |  |  |
| County | 71 (18.8%) | 55 (18.6%) | 16 (19.5%) |  |  |
| Urban | 257 (68.0%) | 204 (68.9%) | 53 (64.6%) |  |  |
| **The only child** |  |  |  | 0.066 | 0.797 |
| No | 277 (73.3%) | 216 (73.0%) | 61 (74.4%) |  |  |
| Yes | 101 (26.7%) | 80 (27.0%) | 21 (25.6%) |  |  |
| **Educational background** |  |  |  | - 0.254 | 0.800 |
| Else | 8 (2.1%) | 7 (2.4%) | 1 (1.2%) |  |  |
| Junior college | 26 (6.9%) | 22 (7.4%) | 4 (4.9%) |  |  |
| Bachelor’s degree | 118 (31.2%) | 91 (30.7%) | 27 (32.9%) |  |  |
| Master’s degree | 95 (25.1%) | 69 (23.3%) | 26 (31.7%) |  |  |
| Doctor’s degree | 131 (24.7%) | 107 (36.1%) | 24 (29.3%) |  |  |
| **Primary family** |  |  |  | 2.185 | 0.344 |
| Nuclear family | 356 (94.2%) | 280 (94.6%) | 76 (92.7%) |  |  |
| Blended family | 12 (3.2%) | 10 (3.4%) | 2 (2.4%) |  |  |
| Single-parent family | 10 (2.6%) | 6 (2.0%) | 4 (4.9%) |  |  |
| **Household income level** |  |  |  | - 1.953 | 0.051 |
| Not good | 23 (6.1%) | 16 (5.4%) | 7 (8.5%) |  |  |
| Not very good | 20 (5.3%) | 11 (3.7%) | 9 (11.0%) |  |  |
| Average | 282 (74.6%) | 226 (76.4%) | 56 (68.3%) |  |  |
| Good | 47 (12.4%) | 38 (12.8%) | 9 (11.0%) |  |  |
| Very good | 6 (1.6%) | 5 (1.7%) | 1 (1.2%) |  |  |
| **Living with parents during 0-3 year-old** |  |  |  | 0.423 | 0.515 |
| No | 56 (14.8%) | 42 (14.2%) | 14 (17.1%) |  |  |
| Yes | 322 (85.2%) | 254 (85.8%) | 68 (82.9%) |  |  |
| **Living with others during quarantine** |  |  |  | 3.519 | 0.061 |
| No | 58 (15.3%) | 40 (13.5%) | 18 (22.0%) |  |  |
| Yes | 320 (84.7%) | 256 (86.5%) | 64 (78.0%) |  |  |
| **Adult attachment style** |  |  |  | 3.537 | 0.060 |
| Secure | 296 (78.3%) | 238 (80.4%) | 58 (70.7%) |  |  |
| Insecure | 82 (21.7%) | 58 (19.6%) | 24 (29.3%) |  |  |
| **Social support rate scale** |  |  |  |  |  |
| Objective support | 11.16$\pm$3.601 | 11.42$\pm$3.475 | 10.23$\pm$3.907 | 2.663 | 0.008 ^*^ |
| Subjective support | 23.97$\pm$4.437 | 24.33$\pm$4.341 | 22.65$\pm$4.555 | 3.083 | 0.002 ^*^ |
| Utilization of support | 7.67$\pm$1.871 | 7.79$\pm$1.821 | 7.23$\pm$1.989 | 2.424 | 0.016 ^*^ |
| **Family cohesion** | 70.98$\pm$11.041 | 72.19$\pm$10.428 | 66.60$\pm$12.105 | 0.132 | $<$0.001 ^*^ |
| **Family adaptability** | 50.59$\pm$9.663 | 51.47$\pm$9.092 | 47.41$\pm$10.978 | 0.016 | $<$0.001 ^*^ |
| **Perceived stress** |  |  |  |  |  |
| **Perceived stress scores** | 5.54$\pm$2.601 | 5.22$\pm$2.544 | 6.68$\pm$2.494 | 0.687 | $<$0.001 ^*^ |
| **Perceived stress – Threshold 8** |  |  |  | 15.591 | $<$0.001 ^*^ |
| Normal | 288 (76.2%) | 239 (80.7%) | 49 (59.8%) |  |  |
| Severe stress | 90 (23.8%) | 57 (19.3%) | 33 (40.2%) |  |  |
| * represents statistical significance. | | | | | |

**Table S3 Univariate analysis of emotional state in doctors**

| **Variables** | **Total**  **(N = 378)** | **Emotional state** | | **t/**$\boldsymbol{\chi}^{\boldsymbol{2}}$**/Z** | ***p* value** |
| --- | --- | --- | --- | --- | --- |
|  |  | **Normal (n = 230)** | **Abnormal (n = 148)** |  |  |
| **Gender** |  |  |  | 2.755 | 0.097 |
| Female | 209 (55.3%) | 135 (58.7%) | 74 (50.0%) |  |  |
| Male | 169 (44.7%) | 95 (41.3%) | 74 (50.0%) |  |  |
| **Age** | 40.75$\pm$10.431 | 41.79$\pm$10.876 | 39.13$\pm$9.511 | 2.506 | 0.013 ^*^ |
| **Permanent address** |  |  |  | 9.559 | 0.008 ^*^ |
| Rural | 50 (13.2%) | 22 (9.6%) | 28 (18.9%) |  |  |
| County | 71 (18.8%) | 51 (22.2%) | 20 (13.5%) |  |  |
| Urban | 257 (68.0%) | 157 (68.3%) | 100 (67.6%) |  |  |
| **The only child** |  |  |  | 0.342 | 0.559 |
| No | 277 (73.3%) | 171 (74.3%) | 109 (71.6%) |  |  |
| Yes | 101 (26.7%) | 59 (25.7%) | 42 (28.4%) |  |  |
| **Educational background** |  |  |  | - 1.298 | 0.194 |
| Else | 8 (2.1%) | 6 (2.6%) | 2 (1.4%) |  |  |
| Junior college | 26 (6.9%) | 17 (7.4%) | 9 (6.1%) |  |  |
| Bachelor’s degree | 118 (31.2%) | 78 (33.9%) | 40 (27.0%) |  |  |
| Master’s degree | 95 (25.1%) | 51 (22.2%) | 44 (29.7%) |  |  |
| Doctor’s degree | 131 (24.7%) | 78 (33.9%) | 53 (35.8%) |  |  |
| **Primary family** |  |  |  | 2.621 | 0.257 |
| Nuclear family | 356 (94.2%) | 220 (95.7%) | 136 (91.9%) |  |  |
| Blended family | 12 (3.2%) | 6 (2.6%) | 6 (4.1%) |  |  |
| Single-parent family | 10 (2.6%) | 4 (1.7%) | 6 (4.1%) |  |  |
| **Household income level** |  |  |  | - 2.417 | 0.016 ^*^ |
| Not good | 23 (6.1%) | 14 (6.1%) | 9 (6.1%) |  |  |
| Not very good | 20 (5.3%) | 6 (2.6%) | 14 (9.5%) |  |  |
| Average | 282 (74.6%) | 172 (74.8%) | 110 (74.3%) |  |  |
| Good | 47 (12.4%) | 33 (14.3%) | 14 (9.5%) |  |  |
| Very good | 6 (1.6%) | 5 (2.2%) | 1 (0.7%) |  |  |
| **Living with parents during 0-3 year-old** |  |  |  | 0.832 | 0.362 |
| No | 56 (14.8%) | 31 (13.5%) | 25 (16.9%) |  |  |
| Yes | 322 (85.2%) | 199 (86.5%) | 123 (83.1%) |  |  |
| **Living with others during quarantine** |  |  |  | 3.383 | 0.066 |
| No | 58 (15.3%) | 29 (12.6%) | 29 (19.6%) |  |  |
| Yes | 320 (84.7%) | 201 (87.4%) | 119 (80.4%) |  |  |
| **Adult attachment style** |  |  |  | 3.107 | 0.078 |
| Secure | 296 (78.3%) | 187 (81.3%) | 109 (73.6%) |  |  |
| Insecure | 82 (21.7%) | 43 (18.7%) | 39 (26.4%) |  |  |
| **Social support rate scale** |  |  |  |  |  |
| Objective support | 11.16$\pm$3.601 | 11.38$\pm$3.573 | 10.82$\pm$3.630 | 1.462 | 0.145 |
| Subjective support | 23.97$\pm$4.437 | 24.79$\pm$4.426 | 22.69$\pm$4.157 | 4.615 | $<$0.001 ^*^ |
| Utilization of support | 7.67$\pm$1.871 | 7.92$\pm$1.842 | 7.28$\pm$1.855 | 3.278 | 0.001 ^*^ |
| **Family cohesion** | 70.98$\pm$11.041 | 72.87$\pm$10.573 | 68.03$\pm$11.139 | 4.270 | $<$0.001 ^*^ |
| **Family adaptability** | 50.59$\pm$9.663 | 52.00$\pm$9.113 | 48.40$\pm$10.107 | 3.592 | $<$0.001 ^*^ |
| **Perceived stress** |  |  |  |  |  |
| **Perceived stress scores** | 5.54$\pm$2.601 | 4.27$\pm$2.277 | 7.51$\pm$1.676 | -15.942 | $<$0.001 ^*^ |
| **Perceived stress – Threshold 8** |  |  |  | 82.727 | $<$0.001 ^*^ |
| Normal | 288 (76.2%) | 212 (92.2%) | 76 (51.4%) |  |  |
| Severe stress | 90 (23.8%) | 18 (7.8%) | 72 (48.6%) |  |  |
| * represents statistical significance. | | | | | |

**Table S4 Univariate analysis of physical health state in doctors**

| **Variables** | **Total**  **(N = 378)** | **Physical health state** | | **t/**$\boldsymbol{\chi}^{\boldsymbol{2}}$**/Z** | ***p* value** |
| --- | --- | --- | --- | --- | --- |
|  |  | **Normal (n = 274)** | **Abnormal (n = 104)** |  |  |
| **Gender** |  |  |  | 0.120 | 0.729 |
| Female | 209 (55.3%) | 150 (54.7%) | 59 (56.7%) |  |  |
| Male | 169 (44.7%) | 124 (45.3%) | 45 (43.3%) |  |  |
| **Age** | 40.75$\pm$10.431 | 41.46$\pm$10.722 | 38.86$\pm$9.412 | 2.313 | 0.022 ^*^ |
| **Permanent address** |  |  |  | 13.722 | 0.001 ^*^ |
| Rural | 50 (13.2%) | 27 (9.9%) | 23 (22.1%) |  |  |
| County | 71 (18.8%) | 60 (21.9%) | 11 (10.6%) |  |  |
| Urban | 257 (68.0%) | 187 (68.2%) | 70 (67.3%) |  |  |
| **The only child** |  |  |  | 0.042 | 0.837 |
| No | 277 (73.3%) | 200 (73.0%) | 77 (74.0%) |  |  |
| Yes | 101 (26.7%) | 74 (27.0%) | 27 (26.0%) |  |  |
| **Educational background** |  |  |  | - 0.960 | 0.337 |
| Else | 8 (2.1%) | 6 (2.2%) | 2 (1.9%) |  |  |
| Junior college | 26 (6.9%) | 19 (6.9%) | 7 (6.7%) |  |  |
| Bachelor’s degree | 118 (31.2%) | 91 (33.2%) | 27 (26.0%) |  |  |
| Master’s degree | 95 (25.1%) | 65 (23.7%) | 30 (28.8%) |  |  |
| Doctor’s degree | 131 (24.7%) | 93 (33.9%) | 38 (36.5%) |  |  |
| **Primary family** |  |  |  | 1.007 | 0.643 |
| Nuclear family | 356 (94.2%) | 259 (94.5%) | 97 (93.3%) |  |  |
| Blended family | 12 (3.2%) | 9 (3.3%) | 3 (2.9%) |  |  |
| Single-parent family | 10 (2.6%) | 6 (2.2%) | 4 (3.8%) |  |  |
| **Household income level** |  |  |  | - 1.251 | 0.211 |
| Not good | 23 (6.1%) | 18 (6.6%) | 5 (4.8%) |  |  |
| Not very good | 20 (5.3%) | 11 (4.0%) | 9 (8.7%) |  |  |
| Average | 282 (74.6%) | 203 (74.1%) | 79 (76.0%) |  |  |
| Good | 47 (12.4%) | 37 (13.5%) | 10 (9.6%) |  |  |
| Very good | 6 (1.6%) | 5 (1.8%) | 1 (1.0%) |  |  |
| **Living with parents during 0-3 year-old** |  |  |  | 3.288 | 0.070 |
| No | 56 (14.8%) | 35 (12.8%) | 21 (20.2%) |  |  |
| Yes | 322 (85.2%) | 239 (87.2%) | 83 (79.8%) |  |  |
| **Living with others during quarantine** |  |  |  | 2.596 | 0.107 |
| No | 58 (15.3%) | 37 (13.5%) | 21 (20.2%) |  |  |
| Yes | 320 (84.7%) | 237 (86.5%) | 83 (79.8%) |  |  |
| **Adult attachment style** |  |  |  | 4.321 | 0.038 ^*^ |
| Secure | 296 (78.3%) | 222 (81.0%) | 74 (71.2%) |  |  |
| Insecure | 82 (21.7%) | 52 (19.0%) | 30 (28.8%) |  |  |
| **Social support rate scale** |  |  |  |  |  |
| Objective support | 11.16$\pm$3.601 | 11.31$\pm$3.606 | 10.78$\pm$3.577 | 1.273 | 0.204 |
| Subjective support | 23.97$\pm$4.437 | 24.42$\pm$4.514 | 22.79$\pm$4.015 | 0.137 | 0.001 ^*^ |
| Utilization of support | 7.67$\pm$1.871 | 7.80$\pm$1.888 | 7.33$\pm$1.787 | 2.221 | 0.027 ^*^ |
| **Family cohesion** | 70.98$\pm$11.041 | 71.87$\pm$11.082 | 68.63$\pm$10.629 | 2.570 | 0.011 ^*^ |
| **Family adaptability** | 50.59$\pm$9.663 | 51.27$\pm$9.860 | 48.81$\pm$8.926 | 2.221 | 0.027 ^*^ |
| **Perceived stress** |  |  |  |  |  |
| **Perceived stress scores** | 5.54$\pm$2.601 | 4.86$\pm$2.511 | 7.32$\pm$1.907 | -10.201 | $<$0.001 ^*^ |
| **Perceived stress – Threshold 8** |  |  |  | 50.341 | $<$0.001 ^*^ |
| Normal | 288 (76.2%) | 235 (85.8%) | 53 (51.0%) |  |  |
| Severe stress | 90 (23.8%) | 39 (14.2%) | 51 (49.0%) |  |  |
| * represents statistical significance. | | | | | |

**Table S5 Univariate analysis of psychosomatic state in doctors**

| **Variables** | **Total** | **Psychosomatic state** | | | **F/**$\boldsymbol{\chi}^{\boldsymbol{2}}$**/H** | ***p* value** |
| --- | --- | --- | --- | --- | --- | --- |
|  |  | **No psychosomatic distress** | **Moderate psychosomatic distress** | **Severe psychosomatic distress** |  |  |
| **Overall** | 378 | 182 (48.1%) | 158 (41.8%) | 38 (10.1%) |  |  |
| **Gender** |  |  |  |  | 3.146 | 0.207 |
| Female | 209 (55.3%) | 100 (54.9%) | 83 (52.5%) | 26 (68.4%) |  |  |
| Male | 169 (44.7%) | 82 (45.1%) | 75 (47.5%) | 12 (31.6%) |  |  |
| **Age** | 40.75$\pm$10.431 | 42.13$\pm$10.924 | 39.70$\pm$10.076 | 34.47$\pm$8.652 | 3.326 | 0.037 ^*^ |
| **Permanent address** |  | **a** |  |  | 12.770 | 0.012 ^*^ |
| Rural | 50 (13.2%) | 14 (7.7%) | 31 (19.6%) | 5 (13.2%) |  |  |
| County | 71 (18.8%) | 42 (23.1%) | 23 (14.6%) | 6 (15.8%) |  |  |
| Urban | 257 (73.6%) | 126 (69.2%) | 104 (65.8%) | 27 (71.1%) |  |  |
| **The only child** |  |  |  |  | 1.191 | 0.551 |
| No | 277 (73.3%) | 131 (72.0%) | 120 (75.9%) | 26 (68.4%) |  |  |
| Yes | 101 (26.7%) | 51 (28.0%) | 38 (24.1%) | 12 (31.6%) |  |  |
| **Educational background** |  |  |  |  | 1.145 | 0.564 |
| Else | 8 (2.1%) | 5 (2.7%) | 3 (1.9%) | 0 (0.0%) |  |  |
| Junior college | 26 (6.9%) | 13 (7.1%) | 12 (7.6%) | 1 (2.6%) |  |  |
| Bachelor’s degree | 118 (31.2%) | 64 (35.2%) | 41 (25.9%) | 13 (34.2%) |  |  |
| Master’s degree | 95 (25.1%) | 37 (20.3%) | 46 (29.1%) | 12 (31.6%) |  |  |
| Doctor’s degree | 131 (34.7%) | 63 (34.6%) | 56 (35.4%) | 12 (31.6%) |  |  |
| **Primary family** |  |  |  |  | 8.688 | 0.047 ^*^ |
| Nuclear family | 356 (94.2%) | 177 (97.3%) | 142 (89.9%) | 37 (97.4%) |  |  |
| Blended family | 12 (3.2%) | 3 (1.6%) | 9 (5.7%) | 0 (0.0%) |  |  |
| Single-parent family | 10 (2.6%) | 2 (1.1%) | 7 (4.4%) | 1 (2.6%) |  |  |
| **Household income level** |  |  |  |  | 6.160 | 0.046 ^*^ |
| Not good | 23 (6.1%) | 12 (6.6%) | 9 (5.7%) | 2 (5.3%) |  |  |
| Not very good | 20 (5.3%) | 4 (2.2%) | 10 (6.3%) | 6 (15.8%) |  |  |
| Average | 282 (74.6%) | 133 (73.1%) | 124 (78.5%) | 25 (65.8%) |  |  |
| Good | 47 (12.4%) | 28 (15.4%) | 15 (9.5%) | 4 (10.5%) |  |  |
| Very good | 6 (1.6%) | 5 (2.7%) | 0 (0.0%) | 1 (2.6%) |  |  |
| **Living with parents during 0-3 year-old** |  |  |  |  | 3.070 | 0.215 |
| No | 56 (14.8%) | 23 (12.6%) | 24 (15.2%) | 9 (23.7%) |  |  |
| Yes | 322 (85.2%) | 159 (87.4%) | 134 (84.8%) | 29 (76.3%) |  |  |
| **Living with others during quarantine** |  | **b** |  |  | 6.443 | 0.040 ^*^ |
| No | 58 (15.3%) | 23 (12.6%) | 24 (15.2%) | 11 (28.9%) |  |  |
| Yes | 320 (84.7%) | 159 (87.4%) | 134 (84.8%) | 27 (71.1%) |  |  |
| **Adult attachment style** |  | **b** |  |  | 6.512 | 0.039 ^*^ |
| Secure | 296 (78.3%) | 149 (81.9%) | 123 (77.8%) | 24 (63.2%) |  |  |
| Insecure | 82 (21.7%) | 33 (18.1%) | 35 (22.2%) | 14 (36.8%) |  |  |
| **Social support rate scale** |  |  |  |  |  |  |
| Objective support | 11.16$\pm$3.601 | 11.55$\pm$3.541 **^b^** | 11.03$\pm$3.478 | 9.84$\pm$4.110 | 3.764 | 0.024 ^*^ |
| Subjective support | 23.97$\pm$4.437 | 25.21$\pm$4.332 **^a, b^** | 23.01$\pm$4.191 | 22.00$\pm$4.343 | 15.641 | $<$0.001 ^*^ |
| Utilization of support | 7.67$\pm$1.871 | 7.99$\pm$1.874 **^a, b^** | 7.49$\pm$1.787 | 6.89$\pm$1.914 | 6.815 | 0.001 ^*^ |
| **Family cohesion** | 70.98$\pm$11.041 | 73.88$\pm$10.499 **^a, b^** | 68.65$\pm$10.487 | 66.74$\pm$12.368 | 13.472 | $<$0.001 ^*^ |
| **Family adaptability** | 50.59$\pm$9.663 | 52.90$\pm$8.736 **^a, b^** | 48.41$\pm$9.957 | 48.58$\pm$10.334 | 10.553 | $<$0.001 ^*^ |
| **Perceived stress** |  |  |  |  |  |  |
| **Perceived stress scores** | 5.54$\pm$2.601 | 4.10$\pm$2.349 **^a, b^** | 6.52$\pm$1.983 **^b^** | 8.32$\pm$1.757 | 88.764 | $<$0.001 ^*^ |
| **Perceived stress – Threshold 8** |  | **a, b** | **b** |  | 79.970 | $<$0.001 ^*^ |
| Normal | 130 (76.2%) | 169 (92.9%) | 108 (68.4%) | 11 (28.9%) |  |  |
| Severe stress | 90 (23.8%) | 13 (7.1%) | 50 (31.6%) | 27 (71.7%) |  |  |
| ^*^ represents statistical significance.  **a** represents statistical difference compared with the moderate psychosomatic distress group, and  **b** represents statistical difference compared with the severe psychosomatic distress group  (after Bonferroni correction for multiple comparisons). | | | | | | |

**Table S6 Multivariate logistic regression analysis of mental health state in doctors**

| **Variables** |  | **Model 1** | |  | **Model 2** | |
| --- | --- | --- | --- | --- | --- | --- |
|  |  | **OR [95% CI]** | ***p* value** |  | **OR [95% CI]** | ***p* value** |
| **Sleep state** | |  |  |  |  |  |
| Gender |  | 0.396 [0.228 0.688] | 0.001 |  | 0.445 [0.258 0.768] | 0.004 |
| Family cohesion |  | 0.965 [0.942 0.988] | 0.003 |  | 0.961 [0.939 0.983] | 0.001 |
| Perceived stress |  | 1.277 [1.140 1.431] | $<$0.001 |  | 2.713 [1.563 4.707] | $<$0.001 |
|  |  |  |  |  |  |  |
| **Emotional state** | |  |  |  |  |  |
| Age |  | 0.965 [0.938 0.993] | 0.013 |  | 0.969 [0.944 0.995] | 0.021 |
| Permanent address |  |  |  |  |  | 0.021 |
| Permanent address (1) |  | - | - |  | 1.613 [0.774 3.361] | 0.202 |
| Permanent address (2) |  | - | - |  | 0.467 [0.235 0.930] | 0.030 |
| Subjective support |  | 0.920 [0.860 0.986] | 0.017 |  | 0.907 [0.855 0.961] | 0.001 |
| Perceived stress |  | 2.376 [1.978 2.855] | $<$0.001 |  | 12.889 [6.884 24.132] | $<$0.001 |
|  |  |  |  |  |  |  |
| **Physical health state** | |  |  |  |  |  |
| Age |  | 0.970 [0.944 0.997] | 0.027 |  | - | - |
| Permanent address |  |  |  |  | 0.002 |  |
| Permanent address (1) |  | 1.703 [0.828 3.502] | 0.148 |  | 2.304 [1.165 4.558] | 0.016 |
| Permanent address (2) |  | 0.448 [0.205 0.978] | 0.044 |  | 0.433 [0.203 0.923] | 0.030 |
| Subjective support |  | - | - |  | 0.925 [0.873 0.980] | 0.008 |
| Perceived stress |  | 1.639 [1.437 1.871] | $<$0.001 |  | 5.569 [3.271 9.481] | $<$0.001 |
| Model 1: Perceived stress score represents perceived stress; Model 2: Perceived stress - threshold 8 represents perceived stress.  OR: Odds ratio; CI: Confidence interval. | | | | | | |

**Table S7 Multivariate logistic regression analysis of psychosomatic health state in doctors**

| **Variables** |  | **Model 1** | |  | **Model 3** | |
| --- | --- | --- | --- | --- | --- | --- |
|  |  | **OR [95% CI]** | ***p* value** |  | **OR [95% CI]** | ***p* value** |
| **No psychosomatic distress vs. Moderate psychosomatic distress** | | | | | | |
| Permanent address |  |  | 0.017 |  |  | 0.002 |
| Permanent address (1) |  | 2.392 [1.086 5.272] | 0.030 |  | 2.738 [1.309 5.726] | 0.007 |
| Permanent address (2) |  | 0.603 [0.304 1.198] | 0.149 |  | 0.528 [0.276 1.013] | 0.055 |
| Primary family |  |  | 0.046 |  |  | 0.022 |
| Primary family (1) |  | 0.314 [0.054 1.815] | 0.196 |  | 0.262 [0.051 1.352] | 0.110 |
| Primary family (2) |  | 1.657 [0.168 16.363] | 0.149 |  | 1.320 [0.158 11.033] | 0.798 |
| Subjective support |  | 0.890 [0.837 0.947] | $<$0.001 |  | 0.885 [0.836 0.937] | $<$0.001 |
| Perceived stress |  | 1.644 [1.441 1.875] | $<$0.001 |  | 6.209 [3.111 12.393] | $<$0.001 |
|  |  |  |  |  |  |  |
| **No psychosomatic distress vs. Severe psychosomatic distress** | | | | | | |
| Age |  | - | - |  | 0.937 [0.888 0.987] | 0.015 |
| Subjective support |  | 0.811 [0.714 0.922] | 0.001 |  | 0.838 [0.749 0.938] | 0.002 |
| Perceived stress |  | 3.027 [2.108 4.437] | $<$0.001 |  | 63.331 [19.234 208.534] | $<$0.001 |
|  |  |  |  |  |  |  |
| **Moderate psychosomatic distress vs. Severe psychosomatic distress** | | | | | | |
| Living with others during quarantine |  | 0.389 [0.157 0.962] | 0.041 |  | 0.399 [0.164 0.973] | 0.043 |
| Perceived stress |  | 1.744 [1.374 2.213] | $<$0.001 |  | 5.500 [2.496 12.122] | $<$0.001 |
| Model 1: Perceived stress score represents perceived stress; Model 2: Perceived stress - threshold 8 represents perceived stress.  OR: Odds ratio; CI: Confidence interval. | | | | | | |

**Table S8 Univariate analysis of sleep state in nurses**

| **Variables** | **Total**  **(N = 244)** | **Sleep state** | | **t/**$\boldsymbol{\chi}^{\boldsymbol{2}}$**/Z** | ***p* value** |
| --- | --- | --- | --- | --- | --- |
|  |  | **Normal (n = 205)** | **Abnormal (n = 39)** |  |  |
| **Gender** |  |  |  | 0.061 | 0.804 |
| Female | 239 (98.0%) | 201 (98.0%) | 38 (97.4%) |  |  |
| Male | 5 (2.0%) | 4 (2.0%) | 1 (2.6%) |  |  |
| **Age** | 36.21$\pm$8.698 | 36.01$\pm$8.415 | 37.28$\pm$10.112 | - 0.837 | 0.404 |
| **Permanent address** |  |  |  | 3.285 | 0.193 |
| Rural | 53 (21.7%) | 47 (22.9%) | 6 (15.4%) |  |  |
| County | 67 (27.5%) | 59 (28.8%) | 8 (20.5%) |  |  |
| Urban | 124 (50.8%) | 99 (48.3%) | 25 (64.1%) |  |  |
| **The only child** |  |  |  | 2.732 | 0.098 |
| No | 171 (70.1%) | 148 (72.2%) | 23 (59.0%) |  |  |
| Yes | 73 (29.9%) | 57 (27.8%) | 16 (41.0%) |  |  |
| **Educational background** |  |  |  | - 0.430 | 0.667 |
| Else | 2 (0.8%) | 2 (1.0%) | 0 (0.0%) |  |  |
| Junior college | 59 (24.2%) | 49 (23.9%) | 10 (25.6%) |  |  |
| Bachelor’s degree | 175 (71.7%) | 146 (71.2%) | 29 (74.4%) |  |  |
| Master’s degree | 8 (3.3%) | 8 (3.9%) | 0 (0.0%) |  |  |
| Doctor’s degree | 0 (0.0%) | 0 (0.0%) | 0 (0.0%) |  |  |
| **Primary family** |  |  |  | 1.799 | 0.495 |
| Nuclear family | 224 (91.8%) | 187 (91.2%) | 37 (94.9%) |  |  |
| Blended family | 10 (4.1%) | 10 (4.9%) | 0 (0.0%) |  |  |
| Single-parent family | 10 (4.1%) | 8 (3.9%) | 2 (5.1%) |  |  |
| **Household income level** |  |  |  | - 0.236 | 0.813 |
| Not good | 7 (2.9%) | 4 (2.0%) | 3 (7.7%) |  |  |
| Not very good | 14 (5.7%) | 12 (5.9%) | 2 (5.1%) |  |  |
| Average | 180 (73.8%) | 154 (75.1%) | 26 (66.7%) |  |  |
| Good | 38 (15.6%) | 30 (14.6%) | 8 (20.5%) |  |  |
| Very good | 5 (2.0%) | 5 (2.4%) | 0 (1.2%) |  |  |
| **Living with parents during 0-3 year-old** |  |  |  | 1.599 | 0.206 |
| No | 45 (18.4%) | 35 (17.1%) | 10 (25.6%) |  |  |
| Yes | 199 (81.6%) | 170 (82.9%) | 29 (74.4%) |  |  |
| **Living with others during quarantine** |  |  |  | 3.732 | 0.053 |
| No | 28 (11.5%) | 20 (9.8%) | 8 (20.8%) |  |  |
| Yes | 216 (88.5%) | 185 (90.2%) | 31 (79.5%) |  |  |
| **Adult attachment style** |  |  |  | 12.835 | $<$0.001 ^*^ |
| Secure | 186 (76.2%) | 165 (80.5%) | 21 (53.8%) |  |  |
| Insecure | 58 (23.8%) | 40 (19.5%) | 18 (46.2%) |  |  |
| **Social support rate scale** |  |  |  |  |  |
| Objective support | 11.38$\pm$4.916 | 11.42$\pm$5.175 | 11.15$\pm$3.265 | 0.314 | 0.753 |
| Subjective support | 24.06$\pm$4.969 | 24.34$\pm$4.871 | 22.56$\pm$5.271 | 2.061 | 0.040 ^*^ |
| Utilization of support | 7.95$\pm$1.864 | 8.01$\pm$1.901 | 7.59$\pm$1.634 | 1.307 | 0.193 |
| **Family cohesion** | 71.37$\pm$11.371 | 71.64$\pm$11.216 | 69.95$\pm$12.211 | 0.850 | 0.396 |
| **Family adaptability** | 51.50$\pm$9.999 | 52.07$\pm$9.948 | 48.54$\pm$9.870 | 2.034 | 0.043 ^*^ |
| **Perceived stress** |  |  |  |  |  |
| **Perceived stress scores** | 5.15$\pm$2.411 | 4.87$\pm$2.319 | 6.59$\pm$2.403 | - 4.213 | $<$0.001 ^*^ |
| **Perceived stress – Threshold 8** |  |  |  | 15.739 | $<$0.001 ^*^ |
| Normal | 199 (81.6%) | 176 (85.9%) | 23 (59.0%) |  |  |
| Severe stress | 45 (18.4%) | 29 (14.1%) | 16 (41.0%) |  |  |
| * represents statistical significance. | | | | | |

**Table S9 Univariate analysis of emotional state in nurses**

| **Variables** | **Total**  **(N = 244)** | **Emotional state** | | **t/**$\boldsymbol{\chi}^{\boldsymbol{2}}$**/Z** | ***p* value** |
| --- | --- | --- | --- | --- | --- |
|  |  | **Normal (n = 183)** | **Abnormal (n = 61)** |  |  |
| **Gender** |  |  |  | 0.068 | 0.794 |
| Female | 239 (98.0%) | 179 (97.8%) | 60 (98.4%) |  |  |
| Male | 5 (2.0%) | 4 (2.2%) | 1 (1.6%) |  |  |
| **Age** | 36.21$\pm$8.698 | 35.96$\pm$8.440 | 36.98$\pm$9.461 | - 0.798 | 0.425 |
| **Permanent address** |  |  |  | 0.343 | 0.842 |
| Rural | 53 (21.7%) | 39 (21.3%) | 14 (23.0%) |  |  |
| County | 67 (27.5%) | 52 (28.4%) | 15 (24.6%) |  |  |
| Urban | 124 (50.8%) | 92 (50.3%) | 34 (52.5%) |  |  |
| **The only child** |  |  |  | 0.163 | 0.687 |
| No | 171 (70.1%) | 127 (69.4%) | 44 (72.1%) |  |  |
| Yes | 73 (29.9%) | 56 (30.6%) | 17 (27.9%) |  |  |
| **Educational background** |  |  |  | - 2.479 | 0.013 ^*^ |
| Else | 2 (0.8%) | 2 (1.1%) | 0 (0.0%) |  |  |
| Junior college | 59 (24.2%) | 50 (27.3%) | 9 (14.8%) |  |  |
| Bachelor’s degree | 175 (71.7%) | 127 (69.4%) | 48 (78.7%) |  |  |
| Master’s degree | 8 (3.3%) | 4 (2.2%) | 4 (6.6%) |  |  |
| Doctor’s degree | 0 (0.0%) | 0 (0.0%) | 0 (0.0%) |  |  |
| **Primary family** |  |  |  | 3.698 | 0.159 |
| Nuclear family | 224 (91.8%) | 171 (93.4%) | 53 (86.9%) |  |  |
| Blended family | 10 (4.1%) | 7 (3.8%) | 3 (4.9%) |  |  |
| Single-parent family | 10 (4.1%) | 5 (2.7%) | 5 (8.2%) |  |  |
| **Household income level** |  |  |  | - 2.236 | 0.025 ^*^ |
| Not good | 7 (2.9%) | 3 (1.6%) | 4 (6.6%) |  |  |
| Not very good | 14 (5.7%) | 9 (4.9%) | 5 (8.2%) |  |  |
| Average | 180 (73.8%) | 135 (73.8%) | 45 (73.8%) |  |  |
| Good | 38 (15.6%) | 31 (16.9%) | 7 (11.5%) |  |  |
| Very good | 5 (2.0%) | 5 (2.7%) | 0 (0.0%) |  |  |
| **Living with parents during 0-3 year-old** |  |  |  | 0.009 | 0.924 |
| No | 45 (18.4%) | 34 (18.6%) | 11 (18.0%) |  |  |
| Yes | 199 (81.6%) | 149 (81.4%) | 50 (82.0%) |  |  |
| **Living with others during quarantine** |  |  |  | 0.215 | 0.643 |
| No | 28 (11.5%) | 20 (10.9%) | 8 (13.1%) |  |  |
| Yes | 216 (88.5%) | 163 (89.1%) | 53 (86.9%) |  |  |
| **Adult attachment style** |  |  |  | 3.649 | 0.056 |
| Secure | 186 (76.2%) | 145 (79.2%) | 41 (67.2%) |  |  |
| Insecure | 58 (23.8%) | 38 (20.8%) | 20 (32.8%) |  |  |
| **Social support rate scale** |  |  |  |  |  |
| Objective support | 11.38$\pm$4.916 | 11.07$\pm$3.420 | 12.33$\pm$7.820 | - 1.744 | 0.082 |
| Subjective support | 24.06$\pm$4.969 | 24.30$\pm$4.941 | 23.34$\pm$5.023 | 1.296 | 0.196 |
| Utilization of support | 7.95$\pm$1.864 | 8.04$\pm$1.910 | 7.66$\pm$1.702 | 1.411 | 0.160 |
| **Family cohesion** | 71.37$\pm$11.371 | 71.99$\pm$11.688 | 69.49$\pm$10.224 | 1.492 | 0.137 |
| **Family adaptability** | 51.50$\pm$9.999 | 52.03$\pm$10.297 | 49.93$\pm$8.944 | 1.419 | 0.157 |
| **Perceived stress** |  |  |  |  |  |
| **Perceived stress scores** | 5.15$\pm$2.411 | 4.46$\pm$2.243 | 7.20$\pm$1.611 | - 8.784 | $<$0.001 ^*^ |
| **Perceived stress – Threshold 8** |  |  |  | 36.048 | $<$0.001 ^*^ |
| Normal | 199 (81.6%) | 165 (90.2%) | 34 (55.7%) |  |  |
| Severe stress | 45 (18.4%) | 18 (9.8%) | 27 (44.3%) |  |  |
| * represents statistical significance. | | | | | |

**Table S10 Univariate analysis of physical health state in nurses**

| **Variables** | **Total**  **(N = 244)** | **Physical health state** | | **t/**$\boldsymbol{\chi}^{\boldsymbol{2}}$**/Z** | ***p* value** |
| --- | --- | --- | --- | --- | --- |
|  |  | **Normal (n = 201)** | **Abnormal (n = 43)** |  |  |
| **Gender** |  |  |  | 1.092 | 0.296 |
| Female | 239 (98.0%) | 196 (97.5%) | 43 (100.0%) |  |  |
| Male | 5 (2.0%) | 5 (2.5%) | 0 (0.0%) |  |  |
| **Age** | 36.21$\pm$8.698 | 35.98$\pm$8.671 | 37.30$\pm$8.844 | - 0.904 | 0.367 |
| **Permanent address** |  |  |  | 2.097 | 0.350 |
| Rural | 53 (21.7%) | 43 (21.4%) | 10 (23.3%) |  |  |
| County | 67 (27.5%) | 59 (29.4%) | 8 (18.6%) |  |  |
| Urban | 124 (50.8%) | 99 (49.3%) | 25 (58.1%) |  |  |
| **The only child** |  |  |  | 0.101 | 0.751 |
| No | 171 (70.1%) | 140 (69.7%) | 31 (72.1%) |  |  |
| Yes | 73 (29.9%) | 61 (30.3%) | 12 (27.9%) |  |  |
| **Educational background** |  |  |  | - 2.081 | 0.037 ^*^ |
| Else | 2 (0.8%) | 2 (1.0%) | 0 (0.0%) |  |  |
| Junior college | 59 (24.2%) | 52 (25.9%) | 7 (16.3%) |  |  |
| Bachelor’s degree | 175 (71.7%) | 143 (71.1%) | 32 (74.4%) |  |  |
| Master’s degree | 8 (3.3%) | 4 (2.0%) | 4 (9.3%) |  |  |
| Doctor’s degree | 0 (0.0%) | 0 (0.0%) | 0 (0.0%) |  |  |
| **Primary family** |  |  |  | 2.053 | 0.255 |
| Nuclear family | 224 (91.8%) | 182 (90.5%) | 42 (97.7%) |  |  |
| Blended family | 10 (4.1%) | 10 (5.0%) | 0 (0.0%) |  |  |
| Single-parent family | 10 (4.1%) | 9 (4.5%) | 1 (2.3%) |  |  |
| **Household income level** |  |  |  | - 1.868 | 0.062 |
| Not good | 7 (2.9%) | 4 (2.0%) | 3 (7.0%) |  |  |
| Not very good | 14 (5.7%) | 9 (4.5%) | 5 (11.6%) |  |  |
| Average | 180 (73.8%) | 151 (75.1%) | 29 (67.4%) |  |  |
| Good | 38 (15.6%) | 33 (16.4%) | 5 (11.6%) |  |  |
| Very good | 5 (2.0%) | 4 (2.0%) | 1 (2.3%) |  |  |
| **Living with parents during 0-3 year-old** |  |  |  | 0.215 | 0.643 |
| No | 45 (18.4%) | 36 (17.9%) | 9 (20.9%) |  |  |
| Yes | 199 (81.6%) | 165 (82.1%) | 34 (79.1%) |  |  |
| **Living with others during quarantine** |  |  |  | 0.243 | 0.622 |
| No | 28 (11.5%) | 24 (11.9%) | 4 (9.3%) |  |  |
| Yes | 216 (88.5%) | 177 (88.1%) | 39 (90.7%) |  |  |
| **Adult attachment style** |  |  |  | 2.225 | 0.136 |
| Secure | 186 (76.2%) | 157 (78.1%) | 29 (67.4%) |  |  |
| Insecure | 58 (23.8%) | 44 (21.9%) | 14 (32.6%) |  |  |
| **Social support rate scale** |  |  |  |  |  |
| Objective support | 11.38$\pm$4.916 | 11.03$\pm$3.444 | 13.02$\pm$8.943 | - 2.438 | 0.015 ^*^ |
| Subjective support | 24.06$\pm$4.969 | 24.11$\pm$5.031 | 23.81$\pm$4.717 | 0.353 | 0.724 |
| Utilization of support | 7.95$\pm$1.864 | 7.93$\pm$1.881 | 8.05$\pm$1.799 | - 0.386 | 0.700 |
| **Family cohesion** | 71.37$\pm$11.371 | 71.22$\pm$11.529 | 72.07$\pm$10.702 | - 0.445 | 0.657 |
| **Family adaptability** | 51.50$\pm$9.999 | 51.29$\pm$10.145 | 52.49$\pm$9.339 | - 0.710 | 0.478 |
| **Perceived stress** |  |  |  |  |  |
| **Perceived stress scores** | 5.15$\pm$2.411 | 4.70$\pm$2.335 | 7.26$\pm$1.465 | -6.896 | $<$0.001 ^*^ |
| **Perceived stress – Threshold 8** |  |  |  | 22.999 | $<$0.001 ^*^ |
| Normal | 199 (76.2%) | 175 (87.1%) | 24 (55.8%) |  |  |
| Severe stress | 45 (18.4%) | 26 (12.9%) | 19 (44.2%) |  |  |
| * represents statistical significance. | | | | | |

**Table S11 Univariate analysis of psychosomatic state in nurses**

| **Variables** | **Total** | **Psychosomatic state** | | | **F/**$\boldsymbol{\chi}^{\boldsymbol{2}}$**/H** | ***p* value** |
| --- | --- | --- | --- | --- | --- | --- |
|  |  | **No psychosomatic distress** | **Moderate psychosomatic distress** | **Severe psychosomatic distress** |  |  |
| **Overall** | 244 | 154 (63.1%) | 80 (32.8%) | 10 (4.1%) |  |  |
| **Gender** |  |  |  |  | 3.446 | 0.171 |
| Female | 171 (70.1%) | 111 (72.1%) | 51 (63.7%) | 9 (90.0%) |  |  |
| Male | 73 (29.9%) | 43 (27.9%) | 29 (36.3%) | 1 (10.0%) |  |  |
| **Age** | 36.21$\pm$8.698 | 35.74$\pm$8.310 **^b^** | 36.25$\pm$8.675 | 43.20$\pm$12.246 | 3.527 | 0.031 ^*^ |
| **Permanent address** |  |  |  |  | 2.243 | 0.704 |
| Rural | 53 (21.7%) | 33 (21.4%) | 19 (23.8%) | 1 (10.0%) |  |  |
| County | 67 (27.5%) | 46 (29.9%) | 19 (23.8%) | 2 (20.0%) |  |  |
| Urban | 124 (50.8%) | 75 (48.7%) | 42 (52.5%) | 7 (70.0%) |  |  |
| **The only child** |  |  |  |  | 3.466 | 0.171 |
| No | 171 (70.1%) | 111 (72.1%) | 51 (63.7%) | 9 (90.0%) |  |  |
| Yes | 73 (29.9%) | 43 (27.9%) | 29 (36.3%) | 1 (10.0%) |  |  |
| **Educational background** |  |  |  |  | 2.444 | 0.295 |
| Else | 2 (0.8%) | 2 (1.3%) | 0 (0.0%) | 0 (0.0%) |  |  |
| Junior college | 59 (24.2%) | 41 (26.6%) | 16 (20.0%) | 2 (20.0%) |  |  |
| Bachelor’s degree | 175 (71.7%) | 107 (69.5%) | 60 (75.0%) | 8 (80.0%) |  |  |
| Master’s degree | 8 (3.3%) | 4 (2.6%) | 4 (5.0%) | 0 (0.0%) |  |  |
| Doctor’s degree | 0 (0.0%) | 0 (0.0%) | 0 (0.0%) | 0 (0.0%) |  |  |
| **Primary family** |  |  |  |  | 1.404 | 0.819 |
| Nuclear family | 224 (91.8%) | 142 (92.2%) | 72 (90.0%) | 10 (100.0%) |  |  |
| Blended family | 10 (4.1%) | 7 (4.5%) | 3 (3.8%) | 0 (0.0%) |  |  |
| Single-parent family | 10 (4.1%) | 5 (3.2%) | 5 (6.3%) | 0 (0.0%) |  |  |
| **Household income level** |  |  |  |  | 3.166 | 0.205 |
| Not good | 7 (2.9%) | 2 (1.3%) | 4 (5.0%) | 1 (10.0%) |  |  |
| Not very good | 14 (5.7%) | 6 (3.9%) | 7 (8.8%) | 1 (10.0%) |  |  |
| Average | 180 (73.8%) | 118 (76.6%) | 55 (68.8%) | 7 (70.0%) |  |  |
| Good | 38 (15.6%) | 24 (15.6%) | 13 (16.3%) | 1 (10.0%) |  |  |
| Very good | 5 (2.0%) | 4 (2.6%) | 1 (1.3%) | 0 (0.0%) |  |  |
| **Living with parents during 0-3 year-old** |  |  |  |  | 0.412 | 0.796 |
| No | 45 (18.4%) | 27 (17.5%) | 16 (20.0%) | 2 (20.0%) |  |  |
| Yes | 199 (81.6%) | 127 (82.5%) | 64 (80.0%) | 8 (80.0%) |  |  |
| **Living with others during quarantine** |  |  |  |  | 3.345 | 0.180 |
| No | 28 (11.5%) | 17 (11.0%) | 8 (10.0%) | 3 (30.0%) |  |  |
| Yes | 216 (88.5%) | 137 (89.0%) | 72 (90.0%) | 7 (70.0%) |  |  |
| **Adult attachment style** |  | **b** |  |  | 9.422 | 0.011 ^*^ |
| Secure | 186 (76.2%) | 124 (80.5%) | 58 (72.5%) | 4 (40.0%) |  |  |
| Insecure | 58 (23.8%) | 30 (19.5%) | 22 (27.5%) | 6 (60.0%) |  |  |
| **Social support rate scale** |  |  |  |  |  |  |
| Objective support | 11.38$\pm$4.916 | 10.99$\pm$3.485 | 12.18$\pm$6.926 | 11.10$\pm$4.067 | 1.562 | 0.212 |
| Subjective support | 24.06$\pm$4.969 | 24.26$\pm$4.905 **^b^** | 24.14$\pm$5.145 | 20.30$\pm$2.946 | 3.048 | 0.049 ^*^ |
| Utilization of support | 7.95$\pm$1.864 | 8.05$\pm$1.936 | 7.80$\pm$1.767 | 7.50$\pm$1.434 | 0.779 | 0.460 |
| **Family cohesion** | 71.37$\pm$11.371 | 71.67$\pm$11.900 | 71.26$\pm$9.879 | 67.60$\pm$14.485 | 0.604 | 0.547 |
| **Family adaptability** | 51.50$\pm$9.999 | 51.81$\pm$10.499 | 51.54$\pm$8.761 | 46.60$\pm$11.128 | 1.276 | 0.281 |
| **Perceived stress** |  |  |  |  |  |  |
| **Perceived stress scores** | 5.15$\pm$2.411 | 4.21$\pm$2.132 **^a, b^** | 6.56$\pm$1.967 **^b^** | 8.30$\pm$1.337 | 46.861 | $<$0.001^*^ |
| **Perceived stress – Threshold 8** |  | **b** |  |  | 47.049 | $<$0.001 ^*^ |
| Normal | 199 (81.6%) | 145 (94.2%) | 50 (62.5%) | 4 (40.0%) |  |  |
| Severe stress | 45 (18.4%) | 9 (5.8%) | 30 (37.5%) | 6 (60.0%) |  |  |
| ^*^ represents statistical significance.  **a** represents statistical difference compared with the moderate psychosomatic distress group, and  **b** represents statistical difference compared with the severe psychosomatic distress group  (after Bonferroni correction for multiple comparisons). | | | | | | |

**Table S12 Multivariate logistic regression analysis of mental health state in nurses**

| **Variables** |  | | **Model 1** | |  | **Model 2** | |
| --- | --- | --- | --- | --- | --- | --- | --- |
|  |  | | **OR [95% CI]** | ***p* value** |  | **OR [95% CI]** | ***p* value** |
| **Sleep state** | |  | |  |  |  |  |
| Adult attachment style |  | | 2.877 [1.368 6.048] | 0.005 |  | 3.049 [1.448 6.420] | 0.003 |
| Perceived stress |  | | 1.361 [1.147 1.614] | $<$0.001 |  | 3.655 [1.687 7.919] | 0.001 |
|  |  | |  |  |  |  |  |
| **Emotional state** | |  | |  |  |  |  |
| Educational background |  | | 3.349 [1.513 7.415] | 0.003 |  | 3.301 [1.559 6.990] | 0.002 |
| Household income level |  | | 0.568 [0.321 1.006] | 0.052 |  | 0.555 [0.329 0.936] | 0.027 |
| Perceived stress |  | | 1.972 [1.612 2.413] | $<$0.001 |  | 8.142 [3.815 17.378] | $<$0.001 |
|  |  | |  |  |  |  |  |
| **Physical health state** | |  | |  |  |  |  |
| Educational background |  | | 3.087 [1.418 6.718] | 0.004 |  | 2.964 [1.434 6.125] | 0.003 |
| Perceived stress |  | | 1.988 [1.627 2.429] | $<$0.001 |  | 8.654 [4.101 18.262] | $<$0.001 |
| Model 1: Perceived stress score represents perceived stress; Model 2: Perceived stress - threshold 8 represents perceived stress.  OR: Odds ratio; CI: Confidence interval. | | | | | | | |

**Table S13 Multivariate logistic regression analysis of psychosomatic health state in nurses**

| **Variables** |  | **Model 1** | |  | **Model 2** | |
| --- | --- | --- | --- | --- | --- | --- |
|  |  | **OR [95% CI]** | ***p* value** |  | **OR [95% CI]** | ***p* value** |
| **No psychosomatic distress vs. Moderate psychosomatic distress** | | | | | | |
| Perceived stress |  | 1.753 [1.477 2.080] | $<$0.001 |  | 9.667 [4.295 21.758] | $<$0.001 |
|  |  |  |  |  |  |  |
| **No psychosomatic distress vs. Severe psychosomatic distress** | | | | | | |
| Age |  | - | - |  | 1.101 [1.014 1.195] | 0.022 |
| Adult attachment style |  | 5.165 [0.945 28.236] | 0.058 |  | - | - |
| Subjective support |  | - | - |  | 0.808 [0.672 0.972] | 0.024 |
| Perceived stress |  | 2.714 [1.665 4.423] | $<$0.001 |  | 20.332 [3.909 105.759] | $<$0.001 |
|  |  |  |  |  |  |  |
| **Moderate psychosomatic distress vs. Severe psychosomatic distress** | | | | | | |
| Age |  | 1.107 [1.022 1.200] | 0.013 |  | 1.105 [1.027 1.188] | 0.007 |
| Subjective support |  | 0.822 [0.697 0.969] | 0.019 |  | 0.805 [0.684 0.946] | 0.009 |
| Perceived stress |  | 1.984 [1.082 3.638] | 0.027 |  | - | - |
| Model 1: Perceived stress score represents perceived stress; Model 2: Perceived stress - threshold 8 represents perceived stress.  OR: Odds ratio; CI: Confidence interval. | | | | | | |

**Table S14 Sociodemographic and psychometric characteristics of male healthcare workers**

| **Variables** | **Total (N = 174)** |  | **Sleep state** | |  | **Emotional state** | |  | **Physical health state** | |
| --- | --- | --- | --- | --- | --- | --- | --- | --- | --- | --- |
|  |  |  | **Normal (n = 148)** | **Abnormal (n = 26)** |  | **Normal (n = 99)** | **Abnormal (n = 75)** |  | **Normal (n = 129)** | **Abnormal (n = 45)** |
| **Age** | 41.71$\pm$11.030 |  | 41.86$\pm$11.217 | 40.81$\pm$10.052 |  | 43.08$\pm$11.561 | 39.89$\pm$10.079 |  | 42.12$\pm$11.456 | 40.51$\pm$9.727 |
| **Permanent address** |  |  |  |  |  |  |  |  |  |  |
| Rural | 23 (13.2%) |  | 20 (13.5%) | 3 (11.5%) |  | 11 (11.1%) | 12 (16.0%) |  | 14 (10.9%) | 9 (20.0%) |
| County | 23 (13.2%) |  | 19 (12.8%) | 4 (15.4%) |  | 16 (16.2%) | 7 (9.3%) |  | 20 (15.5%) | 3 (6.7%) |
| Urban | 128 (73.6%) |  | 109 (73.6%) | 19 (73.1%) |  | 72 (72.7%) | 56 (74.7%) |  | 95 (73.6%) | 33 (73.3%) |
| **The only child** |  |  |  |  |  |  |  |  |  |  |
| No | 133 (76.4%) |  | 112 (75.7%) | 21 (80.8%) |  | 78 (78.8%) | 55 (73.3%) |  | 98 (76.0%) | 35 (77.8%) |
| Yes | 41 (23.6%) |  | 36 (24.3%) | 5 (19.2%) |  | 21 (21.2%) | 20 (26.7%) |  | 31 (24.0%) | 10 (22.2%) |
| **Educational background** |  |  |  |  |  |  |  |  |  |  |
| Else | 4 (2.3%) |  | 4 (2.7%) | 0 (0.0%) |  | 3 (3.0%) | 1 (1.3%) |  | 3 (2.3%) | 1 (2.2%) |
| Junior college | 7 (4.0%) |  | 7 (4.7%) | 0 (0.0%) |  | 5 (5.1%) | 2 (2.7%) |  | 5 (3.9%) | 2 (4.4%) |
| Bachelor’s degree | 40 (23.0%) |  | 35 (23.6%) | 5 (19.2%) |  | 27 (27.3%) | 13 (17.3%) |  | 32 (24.8%) | 8 (17.8%) |
| Master’s degree | 35 (20.1%) |  | 25 (16.9%) | 10 (38.5%) |  | 15 (15.2%) | 20 (26.7%) |  | 24 (18.6%) | 11 (24.4%) |
| Doctor’s degree | 88 (50.6%) |  | 77 (52.0%) | 11 (42.3%) |  | 49 (49.5%) | 39 (52.0%) |  | 65 (50.4%) | 23 (51.1%) |
| **Primary family** |  |  |  |  |  |  |  |  |  |  |
| Nuclear family | 166 (95.4%) |  | 142 (95.9%) | 24 (92.3%) |  | 97 (98.0%) | 69 (92.0%) |  | 123 (95.3%) | 43 (95.6%) |
| Blended family | 3 (1.7%) |  | 2 (1.4%) | 1 (3.8%) |  | 0 (0.0%) | 3 (4.0%) |  | 3 (2.3%) | 0 (0.0%) |
| Single-parent family | 5 (2.9%) |  | 4 (2.7%) | 1 (3.8%) |  | 2 (2.0%) | 3 (4.0%) |  | 3 (2.3%) | 2 (4.4%) |
| **Household income level** |  |  |  |  |  |  |  |  |  |  |
| Not good | 15 (8.6%) |  | 12 (8.1%) | 3 (11.5%) |  | 9 (9.1%) | 6 (8.0%) |  | 12 (9.3%) | 3 (6.7%) |
| Not very good | 14 (8.0%) |  | 10 (6.8%) | 4 (15.4%) |  | 4 (4.0%) | 10 (13.3%) |  | 9 (7.0%) | 5 (11.1%) |
| Average | 123 (70.7%) |  | 107 (72.3%) | 16 (61.5%) |  | 68 (68.7%) | 55 (73.3%) |  | 90 (69.8%) | 33 (73.3%) |
| Good | 17 (9.8%) |  | 14 (9.5%) | 3 (11.5%) |  | 13 (13.1%) | 4 (5.3%) |  | 13 (10.1%) | 4 (8.9%) |
| Very good | 5 (2.9%) |  | 5 (3.4%) | 0 (0.0%) |  | 5 (5.1%) | 0 (0.0%) |  | 5 (3.9%) | 0 (0.0%) |
| **Living with parents during 0-3 year-old** |  |  |  |  |  |  |  |  |  |  |
| No | 23 (13.2%) |  | 17 (11.5%) | 6 (23.1%) |  | 10 (10.1%) | 13 (17.3%) |  | 14 (10.9%) | 9 (20.0%) |
| Yes | 151 (86.8%) |  | 131 (88.5%) | 20 (76.9%) |  | 89 (89.9%) | 62 (82.7%) |  | 115 (89.1%) | 36 (80.0%) |
| **Living with others during quarantine** |  |  |  |  |  |  |  |  |  |  |
| No | 28 (16.1%) |  | 21 (14.2%) | 7 (26.9%) |  | 15 (15.2%) | 13 (17.3%) |  | 20 (15.5%) | 8 (17.8%) |
| Yes | 146 (83.9%) |  | 127 (85.8%) | 19 (73.1%) |  | 84 (84.8%) | 62 (82.7%) |  | 109 (84.5%) | 37 (82.2%) |
| **Adult attachment style** |  |  |  |  |  |  |  |  |  |  |
| Secure | 143 (86.2%) |  | 121 (81.8%) | 22 (84.6%) |  | 85 (85.9%) | 58 (77.3%) |  | 109 (84.5%) | 34 (75.6%) |
| Insecure | 31 (17.8%) |  | 27 (18.2%) | 4 (15.4%) |  | 14 (14.1%) | 17 (22.7%) |  | 20 (15.5%) | 11 (24.4%) |
| **Social support rate scale** |  |  |  |  |  |  |  |  |  |  |
| Objective support | 11.16$\pm$3.600 |  | 11.28$\pm$3.511 | 10.50$\pm$4.082 |  | 11.17$\pm$3.574 | 11.15$\pm$3.657 |  | 11.19$\pm$3.592 | 11.09$\pm$3.661 |
| Subjective support | 24.13$\pm$4.443 |  | 24.24$\pm$4.340 | 23.46$\pm$5.030 |  | 25.12$\pm$4.251 | 22.81$\pm$4.377 |  | 24.50$\pm$4.451 | 23.04$\pm$4.285 |
| Utilization of support | 7.61$\pm$1.934 |  | 7.66$\pm$1.887 | 7.38$\pm$2.210 |  | 7.93$\pm$1.976 | 7.20$\pm$1.808 |  | 7.79$\pm$1.991 | 7.11$\pm$1.682 |
| **Family cohesion** | 71.26$\pm$9.630 |  | 71.61$\pm$9.456 | 69.23$\pm$10.531 |  | 72.86$\pm$9.400 | 69.15$\pm$9.583 |  | 72.07$\pm$9.748 | 68.93$\pm$8.986 |
| **Family adaptability** | 51.14$\pm$8.528 |  | 51.40$\pm$8.466 | 49.65$\pm$8.895 |  | 52.67$\pm$8.022 | 49.12$\pm$8.806 |  | 51.68$\pm$8.961 | 49.58$\pm$6.998 |
| **Perceived stress** |  |  |  |  |  |  |  |  |  |  |
| **Perceived stress scores** | 5.84$\pm$2.475 |  | 5.64$\pm$2.450 | 6.96$\pm$2.358 |  | 4.65$\pm$2.287 | 7.41$\pm$1.725 |  | 5.33$\pm$2.479 | 7.31$\pm$1.794 |
| **Perceived stress – Threshold 8** |  |  |  |  |  |  |  |  |  |  |
| Normal | 130 (74.7%) |  | 116 (78.4%) | 14 (53.8%) |  | 91 (91.9%) | 39 (52.0%) |  | 108 (83.7%) | 22 (48.9%) |
| Severe stress | 44 (25.3%) |  | 32 (21.6%) | 12 (46.2%) |  | 8 (8.1%) | 36 (48.0%) |  | 21 (16.3%) | 23 (51.1%) |

**Table S15 Univariate analysis of mental health in male healthcare workers**

| **Variables** | **Sleep state** | |  | **Emotional state** | |  | **Physical health state** | |
| --- | --- | --- | --- | --- | --- | --- | --- | --- |
|  | **t/**$\boldsymbol{\chi}^{\boldsymbol{2}}$**/Z** | ***p* value** |  | **t/**$\boldsymbol{\chi}^{\boldsymbol{2}}$**/Z** | ***p* value** |  | **t/**$\boldsymbol{\chi}^{\boldsymbol{2}}$**/Z** | ***p* value** |
| **Age** | 0.450 | 0.654 |  | 1.938 | 0.054 |  | 0.844 | 0.400 |
| **Permanent address** | 0.278 | 0.940 |  | 2.299 | 0.317 |  | 4.083 | 0.130 |
| **The only child** | 0.319 | 0.572 |  | 0.705 | 0.401 |  | 0.061 | 0.806 |
| **Educational background** | - 0.037 | 0.971 |  | - 1.104 | 0.270 |  | - 0.348 | 0.727 |
| **Primary family** | 1.876 | 0.341 |  | 4.328 | 0.111 |  | 1.316 | 0.553 |
| **Household income level** | - 1.167 | 0.243 |  | - 2.424 | 0.015^*^ |  | - 0.659 | 0.510 |
| **Living with parents during 0-3 year-old** | 1.678 | 0.195 |  | 1.946 | 0.163 |  | 2.433 | 0.119 |
| **Living with others during quarantine** | 1.796 | 0.180 |  | 0.150 | 0.698 |  | 0.128 | 0.721 |
| **Adult attachment style** | 0.005 | 0.941 |  | 2.118 | 0.146 |  | 1.821 | 0.177 |
| **Social support rate scale** |  |  |  |  |  |  |  |  |
| Objective support | 1.015 | 0.311 |  | 0.045 | 0.964 |  | 0.155 | 0.877 |
| Subjective support | 0.827 | 0.410 |  | 3.502 | $<$0.001^*^ |  | 1.912 | 0.058 |
| Utilization of support | 0.657 | 0.512 |  | 2.500 | 0.013^*^ |  | 2.222 | 0.029^*^ |
| **Family cohesion** | 1.165 | 0.245 |  | 2.558 | 0.011^*^ |  | 1.895 | 0.060 |
| **Family adaptability** | 0.962 | 0.337 |  | 2.769 | 0.006^*^ |  | 1.430 | 0.155 |
| **Perceived stress** |  |  |  |  |  |  |  |  |
| **Perceived stress scores** | - 2.547 | 0.012^*^ |  | - 9.097 | $<$0.001^*^ |  | 0.033 | $<$0.001^*^ |
| **Perceived stress – Threshold 8** | 7.045 | 0.008^*^ |  | 35.993 | $<$0.001^*^ |  | 21.425 | $<$0.001^*^ |
| * represents statistical significance. | | | | | | | | |

**Table S16 Univariate analysis of** **psychosomatic state in male healthcare workers**

| **Variables** | **Total** | **Psychosomatic state** | | | **F/**$\boldsymbol{\chi}^{\boldsymbol{2}}$**/H** | ***p* value** |
| --- | --- | --- | --- | --- | --- | --- |
|  |  | **No psychosomatic distress** | **Moderate psychosomatic distress** | **Severe psychosomatic distress** |  |  |
| **Overall** | 174 | 85 (48.9%) | 77 (44.3%) | 12 (6.9%) |  |  |
| **Age** | 41.71$\pm$11.030 | 43.54$\pm$11.546 | 40.03$\pm$10.621 | 39.50$\pm$8.062 | 2.346 | 0.099 |
| **Permanent address** |  |  |  |  | 3.261 | 0.488 |
| Rural | 23 (13.2%) | 9 (10.6%) | 13 (16.9%) | 1 (8.3%) |  |  |
| County | 23 (13.2%) | 14 (16.5%) | 7 (9.1%) | 2 (16.7%) |  |  |
| Urban | 128 (73.6%) | 62 (72.9%) | 57 (74.0%) | 9 (75.0%) |  |  |
| **The only child** |  |  |  |  | 1.047 | 0.639 |
| No | 133 (76.4%) | 67 (78.8%) | 56 (72.7%) | 10 (83.3%) |  |  |
| Yes | 41 (23.6%) | 18 (21.2%) | 21 (27.3%) | 2 (16.7%) |  |  |
| **Educational background** |  |  |  |  | 0.421 | 0.810 |
| Else | 4 (2.3%) | 3 (3.5%) | 1 (1.3%) | 0 (0.0%) |  |  |
| Junior college | 7 (4.0%) | 5 (5.9%) | 2 (2.6%) | 0 (0.0%) |  |  |
| Bachelor’s degree | 40 (23.0%) | 22 (25.9%) | 16 (20.8%) | 2 (16.7%) |  |  |
| Master’s degree | 35 (20.1%) | 11 (12.9%) | 19 (24.7%) | 5 (41.7%) |  |  |
| Doctor’s degree | 88 (50.6%) | 44 (51.8%) | 39 (50.6%) | 5 (41.7%) |  |  |
| **Primary family** |  |  |  |  | 5.600 | 0.186 |
| Nuclear family | 166 (95.4%) | 84 (98.8%) | 70 (90.9%) | 12 (100.0%) |  |  |
| Blended family | 3 (1.7%) | 0 (0.0%) | 3 (3.9%) | 0 (0.0%) |  |  |
| Single-parent family | 5 (2.9%) | 1 (1.2%) | 4 (5.2%) | 0 (0.0%) |  |  |
| **Household income level** |  |  |  |  | 3.930 | 0.140 |
| Not good | 15 (8.6%) | 9 (10.6%) | 6 (7.8%) | 0 (0.0%) |  |  |
| Not very good | 14 (8.0%) | 3 (3.5%) | 8 (10.4%) | 3 (25.0%) |  |  |
| Average | 123 (70.7%) | 57 (67.1%) | 59 (76.6%) | 7 (58.3%) |  |  |
| Good | 17 (9.8%) | 11 (12.9%) | 4 (5.2%) | 2 (16.7%) |  |  |
| Very good | 5 (2.9%) | 5 (5.9%) | 0 (0.0%) | 0 (0.0%) |  |  |
| **Living with parents during 0-3 year-old** |  |  |  |  | 4.940 | 0.063 |
| No | 23 (13.2%) | 8 (9.4%) | 11 (14.3%) | 4 (33.3%) |  |  |
| Yes | 151 (86.8%) | 77 (90.6%) | 66 (85.7%) | 8 (66.7%) |  |  |
| **Living with others during quarantine** |  |  |  |  | 5.482 | 0.056 |
| No | 28 (16.1%) | 13 (15.3%) | 10 (13.0%) | 9 (41.7%) |  |  |
| Yes | 146 (83.9%) | 72 (84.7%) | 67 (87.0%) | 7 (58.3%) |  |  |
| **Adult attachment style** |  |  |  |  | 2.434 | 0.314 |
| Secure | 143 (82.2%) | 72 (84.7%) | 63 (81.8%) | 8 (66.7%) |  |  |
| Insecure | 31 (17.8%) | 13 (15.3%) | 14 (18.2%) | 4 (33.3%) |  |  |
| **Social support rate scale** |  |  |  |  |  |  |
| Objective support | 11.16$\pm$3.600 | 11.18$\pm$3.533 | 11.42$\pm$3.507 | 9.42$\pm$4.441 | 1.614 | 0.202 |
| Subjective support | 24.13$\pm$4.443 | 25.25$\pm$4.220 **^a, b^** | 23.26$\pm$4.315 | 21.75$\pm$4.975 | 6.242 | 0.002^*^ |
| Utilization of support | 7.61$\pm$1.934 | 8.01$\pm$2.015 | 7.30$\pm$1.740 | 6.83$\pm$2.082 | 3.926 | 0.022^*^ |
| **Family cohesion** | 71.26$\pm$9.630 | 72.87$\pm$9.497 | 70.19$\pm$9.445 | 66.67$\pm$10.120 | 3.098 | 0.048^*^ |
| **Family adaptability** | 51.14$\pm$8.528 | 52.80$\pm$8.160 | 49.68$\pm$8.820 | 48.75$\pm$7.424 | 3.303 | 0.039^*^ |
| **Perceived stress** |  |  |  |  |  |  |
| **Perceived stress scores** | 5.84$\pm$2.475 | 4.55$\pm$2.327 **^a, b^** | 6.91$\pm$1.928 | 8.08$\pm$1.730 | 32.101 | $<$0.001^*^ |
| **Perceived stress – Threshold 8** |  | **a, b** |  |  | 33.348 | $<$0.001^*^ |
| Normal | 130 (74.7%) | 78 (91.8%) | 49 (63.6%) | 3 (25.0%) |  |  |
| Severe stress | 44 (25.3%) | 7 (8.2%) | 28 (36.4%) | 9 (75.0%) |  |  |
| ^*^ represents statistical significance.  **a** represents statistical difference compared with the moderate psychosomatic distress group, and  **b** represents statistical difference compared with the severe psychosomatic distress group  (after Bonferroni correction for multiple comparisons). | | | | | | |

**Table S17** **Multivariate logistic regression analysis of mental health state in male healthcare workers**

| **Variables** |  | **Model 1** | |  | **Model 2** | |
| --- | --- | --- | --- | --- | --- | --- |
|  |  | **OR [95% CI]** | ***p* value** |  | **OR [95% CI]** | ***p* value** |
| **Sleep state** | |  |  |  |  |  |
| Perceived stress |  | 1.280 [1.051 1.558] | 0.014 |  | 3.107 [1.309 7.377] | 0.010 |
|  |  |  |  |  |  |  |
| **Emotional state** | |  |  |  |  |  |
| Subjective support |  | 0.901 [0.824 0.985] | 0.023 |  | 0.884 [0.816 0.959] | 0.003 |
| Perceived stress |  | 1.999 [1.591 2.511] | $<$0.001 |  | 10.480 [4.363 25.177] | $<$0.001 |
|  |  |  |  |  |  |  |
| **Physical health state** | |  |  |  |  |  |
| Perceived stress |  | 1.509 [1.254 1.816] | $<$0.001 |  | 5.377 [2.544 11.364] | $<$0.001 |
| Model 1: Perceived stress score represents perceived stress; Model 2: Perceived stress - threshold 8 represents perceived stress.  OR: Odds ratio; CI: Confidence interval. | | | | | | |

**Table S18 Multivariate logistic regression analysis of** **psychosomatic health state in male healthcare workers**

| **Variables** |  | **Model 1** | |  | **Model 3** | |
| --- | --- | --- | --- | --- | --- | --- |
|  |  | **OR [95% CI]** | ***p* value** |  | **OR [95% CI]** | ***p* value** |
| **No psychosomatic distress vs. Moderate psychosomatic distress** | | | | | | |
| Subjective support |  | - | - |  | 0.902 [0.834 0.977] | 0.011 |
| Perceived stress |  | 1.692 [1.398 2.048] | $<$0.001 |  | 6.081 [2.432 15.201] | $<$0.001 |
|  |  |  |  |  |  |  |
| **No** **psychosomatic distress vs. Severe psychosomatic distress** | | | | | | |
| Subjective support |  | 0.767 [0.611 0.962] | 0.022 |  | 0.772 [0.624 0.954] | 0.017 |
| Perceived stress |  | 3.336 [1.719 6.475] | $<$0.001 |  | 56.121 [8.403 374.837] | $<$0.001 |
|  |  |  |  |  |  |  |
| **Moderate psychosomatic distress vs. Severe psychosomatic distress** | | | | | | |
| Perceived stress |  | 1.432 [0.991 2.069] | 0.056 |  | 5.250 [1.312 21.008] | 0.019 |
| Model 1: Perceived stress score represents perceived stress; Model 2: Perceived stress - threshold 8 represents perceived stress.  OR: Odds ratio; CI: Confidence interval. | | | | | | |

**Table S19 Sociodemographic and psychometric characteristics of female healthcare workers**

| **Variables** | **Total (N = 448)** |  | **Sleep state** | |  | **Emotional state** | |  | **Physical health state** | |
| --- | --- | --- | --- | --- | --- | --- | --- | --- | --- | --- |
|  |  |  | **Normal (n = 353)** | **Abnormal (n = 95)** |  | **Normal (n = 314)** | **Abnormal (n = 134)** |  | **Normal (n = 346)** | **Abnormal (n = 102)** |
| **Age** | 37.90$\pm$9.411 |  | 37.78$\pm$9.270 | 38.37$\pm$9.957 |  | 37.98$\pm$9.536 | 37.72$\pm$9.146 |  | 38.03$\pm$9.561 | 37.47$\pm$8.917 |
| **Permanent address** |  |  |  |  |  |  |  |  |  |  |
| Rural | 80 (17.9%) |  | 64 (18.1%) | 16 (16.8%) |  | 50 (15.9%) | 30 (22.4%) |  | 56 (16.2%) | 24 (23.5%) |
| County | 115 (25.7%) |  | 95 (26.9%) | 20 (21.1%) |  | 87 (27.7%) | 28 (20.9%) |  | 99 (28.6%) | 16 (15.7%) |
| Urban | 253 (56.5%) |  | 194 (55.0%) | 59 (62.1%) |  | 177 (56.4%) | 76 (56.7%) |  | 191 (55.2%) | 62 (60.8%) |
| **The only child** |  |  |  |  |  |  |  |  |  |  |
| No | 315 (70.3%) |  | 252 (71.4%) | 63 (66.3%) |  | 220 (70.1%) | 95 (70.9%) |  | 242 (69.9%) | 73 (71.6%) |
| Yes | 133 (29.7%) |  | 101 (28.6%) | 32 (33.7%) |  | 94 (29.9%) | 39 (29.1%) |  | 104 (30.1%) | 29 (28.4%) |
| **Educational background** |  |  |  |  |  |  |  |  |  |  |
| Else | 6 (1.3%) |  | 5 (1.4%) | 1 (1.1%) |  | 5 (1.6%) | 1 (0.7%) |  | 5 (1.4%) | 1 (1.0%) |
| Junior college | 78 (17.4%) |  | 64 (18.1%) | 14 (14.7%) |  | 62 (19.7%) | 16 (11.9%) |  | 66 (19.1%) | 12 (11.8%) |
| Bachelor’s degree | 253 (56.5%) |  | 202 (57.2%) | 51 (53.7%) |  | 178 (56.7%) | 75 (56.0%) |  | 202 (58.4%) | 51 (50.0%) |
| Master’s degree | 68 (15.2%) |  | 52 (14.7%) | 16 (16.8%) |  | 40 (12.7%) | 28 (20.9%) |  | 45 (13.0%) | 23 (22.5%) |
| Doctor’s degree | 43 (9.6%) |  | 30 (8.5%) | 13 (13.7%) |  | 29 (9.2%) | 14 (10.4%) |  | 28 (8.1%) | 15 (14.7%) |
| **Primary family** |  |  |  |  |  |  |  |  |  |  |
| Nuclear family | 414 (92.4%) |  | 325 (92.1%) | 89 (93.7%) |  | 294 (93.6%) | 120 (89.6%) |  | 318 (91.9%) | 96 (94.1%) |
| Blended family | 19 (4.2%) |  | 18 (5.1%) | 1 (1.1%) |  | 13 (4.1%) | 6 (4.5%) |  | 16 (4.6%) | 3 (2.9%) |
| Single-parent family | 15 (3.3%) |  | 10 (2.8%) | 5 (5.3%) |  | 7 (2.2%) | 8 (6.0%) |  | 12 (3.5%) | 3 (2.9%) |
| **Household income level** |  |  |  |  |  |  |  |  |  |  |
| Not good | 15 (3.3%) |  | 8 (2.3%) | 7 (7.4%) |  | 8 (2.5%) | 7 (5.2%) |  | 10 (2.9%) | 5 (4.9%) |
| Not very good | 20 (4.5%) |  | 13 (3.7%) | 7 (7.4%) |  | 11 (3.5%) | 9 (6.7%) |  | 1 (3.2%) | 9 (8.8%) |
| Average | 339 (75.7%) |  | 273 (77.3%) | 66 (69.5%) |  | 239 (76.1%) | 100 (74.6%) |  | 264 (76.3%) | 75 (73.5%) |
| Good | 68 (15.2%) |  | 54 (15.3%) | 14 (14.7%) |  | 51 (16.2%) | 17 (12.7%) |  | 57 (16.5%) | 11 (10.8%) |
| Very good | 6 (1.3%) |  | 5 (1.4%) | 1 (1.1%) |  | 5 (1.6%) | 1 (0.7%) |  | 4 (1.2%) | 2 (2.0%) |
| **Living with parents during 0-3 year-old** |  |  |  |  |  |  |  |  |  |  |
| No | 78 (17.4%) |  | 60 (17.0%) | 18 (18.9%) |  | 55 (17.5%) | 23 (17.2%) |  | 57 (16.5%) | 21 (20.6%) |
| Yes | 370 (82.6%) |  | 293 (83.0%) | 77 (81.1%) |  | 259 (82.5%) | 111 (82.8%) |  | 289 (83.5%) | 81 (79.4%) |
| **Living with others during quarantine** |  |  |  |  |  |  |  |  |  |  |
| No | 58 (12.9%) |  | 39 (11.0%) | 19 (20.0%) |  | 34 (10.8%) | 24 (17.9%) |  | 41 (11.8%) | 17 (16.7%) |
| Yes | 390 (87.1%) |  | 314 (89.0%) | 76 (80.0%) |  | 280 (89.2%) | 110 (82.1%) |  | 305 (88.2%) | 85 (83.3%) |
| **Adult attachment style** |  |  |  |  |  |  |  |  |  |  |
| Secure | 339 (75.7%) |  | 282 (79.9%) | 57 (60.0%) |  | 247 (78.7%) | 92 (68.7%) |  | 270 (78.0%) | 69 (67.6%) |
| Insecure | 109 (24.3%) |  | 71 (20.1%) | 38 (40.0%) |  | 67 (21.3%) | 42 (31.3%) |  | 76 (22.0%) | 33 (32.4%) |
| **Social support rate scale** |  |  |  |  |  |  |  |  |  |  |
| Objective support | 11.28$\pm$4.367 |  | 11.48$\pm$4.526 | 10.54$\pm$3.643 |  | 11.26$\pm$3.489 | 11.33$\pm$5.953 |  | 11.19$\pm$3.522 | 11.59$\pm$6.474 |
| Subjective support | 23.96$\pm$4.731 |  | 24.38$\pm$4.655 | 22.39$\pm$4.705 |  | 24.40$\pm$4.778 | 22.92$\pm$4.467 |  | 24.21$\pm$4.843 | 23.11$\pm$4.245 |
| Utilization of support | 7.84$\pm$1.845 |  | 7.98$\pm$1.836 | 7.34$\pm$1.796 |  | 7.99$\pm$1.839 | 7.50$\pm$1.818 |  | 7.88$\pm$1.845 | 7.73$\pm$1.846 |
| **Family cohesion** | 71.08$\pm$11.716 |  | 72.11$\pm$11.257 | 67.25$\pm$12.624 |  | 72.37$\pm$11.565 | 68.07$\pm$11.553 |  | 71.42$\pm$11.790 | 69.94$\pm$11.442 |
| **Family adaptability** | 50.87$\pm$10.258 |  | 51.85$\pm$9.837 | 47.26$\pm$11.014 |  | 51.81$\pm$10.104 | 48.69$\pm$10.323 |  | 51.13$\pm$10.331 | 50.02$\pm$10.010 |
| **Perceived stress** |  |  |  |  |  |  |  |  |  |  |
| **Perceived stress scores** | 5.21$\pm$2.536 |  | 4.84$\pm$2.426 | 6.57$\pm$2.487 |  | 4.26$\pm$2.249 | 7.43$\pm$1.629 |  | 4.59$\pm$2.394 | 7.29$\pm$1.789 |
| **Perceived stress – Threshold 8** |  |  |  |  |  |  |  |  |  |  |
| Normal | 357 (79.7%) |  | 299 (84.7%) | 58 (61.1%) |  | 286 (91.1%) | 71 (53.0%) |  | 302 (87.3%) | 55 (53.9%) |
| Severe stress | 91 (20.3%) |  | 54 (15.3%) | 37 (38.9%) |  | 28 (8.9%) | 63 (47.0%) |  | 44 (12.7%) | 47 (46.1%) |

**Table S20 Univariate analysis of mental health in female healthcare workers**

| **Variables** | **Sleep state** | |  | **Emotional state** | |  | **Physical health state** | |
| --- | --- | --- | --- | --- | --- | --- | --- | --- |
|  | **t/**$\boldsymbol{\chi}^{\boldsymbol{2}}$**/Z** | ***p* value** |  | **t/**$\boldsymbol{\chi}^{\boldsymbol{2}}$**/Z** | ***p* value** |  | **t/**$\boldsymbol{\chi}^{\boldsymbol{2}}$**/Z** | ***p* value** |
| **Age** | - 0.541 | 0.589 |  | 0.264 | 0.792 |  | 0.529 | 0.597 |
| **Permanent address** | 1.748 | 0.417 |  | 3.897 | 0.142 |  | 7.942 | 0.019^*^ |
| **The only child** | 0.923 | 0.337 |  | 0.031 | 0.860 |  | 0.100 | 0.752 |
| **Educational background** | - 1.556 | 0.120 |  | - 2.527 | 0.012^*^ |  | - 3.249 | 0.001^*^ |
| **Primary family** | 4.335 | 0.121 |  | 4.003 | 0.134 |  | 0.435 | 0.850 |
| **Household income level** | - 1.664 | 0.096 |  | - 2.026 | 0.043^*^ |  | - 2.169 | 0.030^*^ |
| **Living with parents during 0-3 year-old** | 0.198 | 0.656 |  | 0.008 | 1.000 |  | 0.927 | 0.336 |
| **Living with others during quarantine** | 5.322 | 0.021^*^ |  | 4.180 | 0.041^*^ |  | 1.622 | 0.203 |
| **Adult attachment style** | 16.080 | $<$0.001^*^ |  | 5.107 | 0.024^*^ |  | 4.617 | 0.032^*^ |
| **Social support rate scale** |  |  |  |  |  |  |  |  |
| Objective support | 1.877 | 0.061 |  | - 0.149 | 0.882 |  | - 0.808 | 0.420 |
| Subjective support | 3.685 | $<$0.001^*^ |  | 3.060 | 0.002^*^ |  | 2.066 | 0.039^*^ |
| Utilization of support | 3.045 | 0.002^*^ |  | 2.593 | 0.010^*^ |  | 0.736 | 0.462 |
| **Family cohesion** | 3.636 | $<$0.001^*^ |  | 3.604 | $<$0.001^*^ |  | 1.118 | 0.264 |
| **Family adaptability** | 3.928 | $<$0.001^*^ |  | 2.965 | 0.003^*^ |  | 0.958 | 0.338 |
| **Perceived stress** |  |  |  |  |  |  |  |  |
| **Perceived stress scores** | - 6.127 | $<$0.001^*^ |  | - 16.701 | $<$0.001^*^ |  | - 12.339 | $<$0.001^*^ |
| **Perceived stress – Threshold 8** | 25.866 | $<$0.001^*^ |  | 84.217 | $<$0.001^*^ |  | 54.168 | $<$0.001^*^ |
| * represents statistical significance. | | | | | | | | |

**Table S21 Univariate analysis of psychosomatic state in female healthcare workers**

| **Variables** | **Total** | **Psychosomatic state** | | | **F/**$\boldsymbol{\chi}^{\boldsymbol{2}}$**/H** | ***p* value** |
| --- | --- | --- | --- | --- | --- | --- |
|  |  | **No psychosomatic distress** | **Moderate psychosomatic distress** | **Severe psychosomatic distress** |  |  |
| **Overall** | 448 | 251 (56.0%) | 161 (35.9%) | 36 (8.0%) |  |  |
| **Age** | 37.90$\pm$9.411 | 37.73$\pm$9.428 | 37.83$\pm$9.252 | 39.44$\pm$10.115 | 0.529 | 0.589 |
| **Permanent address** |  |  |  |  | 8.416 | 0.077 |
| Rural | 80 (17.9%) | 38 (15.1%) | 37 (23.0%) | 5 (13.9%) |  |  |
| County | 115 (25.7%) | 74 (29.5%) | 35 (21.7%) | 6 (16.7%) |  |  |
| Urban | 253 (56.5%) | 139 (55.4%) | 89 (55.3%) | 25 (69.4%) |  |  |
| **The only child** |  |  |  |  | 0.151 | 0.927 |
| No | 315 (70.3%) | 175 (69.7%) | 115 (71.4%) | 25 (69.4%) |  |  |
| Yes | 133 (29.7%) | 76 (30.3%) | 46 (28.6%) | 11 (30.6%) |  |  |
| **Educational background** |  | **b** |  |  | 10.408 | 0.005^*^ |
| Else | 6 (1.3%) | 4 (1.6%) | 2 (1.2%) | 0 (0.0%) |  |  |
| Junior college | 78 (17.4%) | 49 (19.5%) | 26 (16.1%) | 3 (8.3%) |  |  |
| Bachelor’s degree | 253 (56.5%) | 149 (59.4%) | 85 (52.8%) | 19 (52.8%) |  |  |
| Master’s degree | 68 (15.2%) | 30 (12.0%) | 31 (19.3%) | 7 (19.4%) |  |  |
| Doctor’s degree | 43 (9.6%) | 19 (7.6%) | 17 (10.6%) | 7 (19.4%) |  |  |
| **Primary family** |  |  |  |  | 3.985 | 0.370 |
| Nuclear family | 414 (92.4%) | 235 (93.6%) | 144 (89.4%) | 35 (97.2%) |  |  |
| Blended family | 19 (4.2%) | 10 (4.0%) | 9 (5.6%) | 0 (0.0%) |  |  |
| Single-parent family | 15 (3.3%) | 6 (2.4%) | 8 (5.0%) | 1 (2.8%) |  |  |
| **Household income level** |  |  |  |  | 6.617 | 0.037^*^ |
| Not good | 15 (3.3%) | 5 (2.0%) | 7 (4.3%) | 3 (8.3%) |  |  |
| Not very good | 20 (4.5%) | 7 (2.8%) | 9 (5.6%) | 4 (11.1%) |  |  |
| Average | 339 (75.7%) | 194 (77.3%) | 120 (74.5%) | 25 (69.4%) |  |  |
| Good | 68 (15.2%) | 41 (16.3%) | 24 (14.9%) | 3 (8.3%) |  |  |
| Very good | 6 (1.3%) | 4 (1.6%) | 1 (0.6%) | 1 (2.8%) |  |  |
| **Living with parents during 0-3 year-old** |  |  |  |  | 0.224 | 0.894 |
| No | 78 (17.4%) | 42 (16.7%) | 29 (18.0%) | 7 (19.4%) |  |  |
| Yes | 370 (82.6%) | 209 (83.3%) | 132 (82.0%) | 29 (80.6%) |  |  |
| **Living with others during quarantine** |  |  |  |  | 5.419 | 0.065 |
| No | 58 (12.9%) | 27 (10.8%) | 22 (13.7%) | 9 (25.0%) |  |  |
| Yes | 390 (87.1%) | 224 (89.2%) | 139 (86.3%) | 27 (75.0%) |  |  |
| **Adult attachment style** |  | **b** |  |  | 11.057 | 0.004^*^ |
| Secure | 339 (75.7%) | 201 (80.1%) | 118 (73.3%) | 20 (55.6%) |  |  |
| Insecure | 109 (24.3%) | 50 (19.9%) | 43 (26.7%) | 16 (44.4%) |  |  |
| **Social support rate scale** |  |  |  |  |  |  |
| Objective support | 11.28$\pm$4.367 | 11.33$\pm$3.523 | 11.42$\pm$5.490 | 10.33$\pm$4.007 | 0.941 | 0.391 |
| Subjective support | 23.96$\pm$4.731 | 24.61$\pm$4.746 **^a, b^** | 23.45$\pm$4.677 | 21.61$\pm$3.879 | 7.997 | $<$0.001^*^ |
| Utilization of support | 7.84$\pm$1.845 | 8.02$\pm$1.864 **^b^** | 7.74$\pm$1.791 | 7.08$\pm$1.763 | 4.533 | 0.011^*^ |
| **Family cohesion** | 71.08$\pm$11.716 | 72.87$\pm$11.739 **^a, b^** | 69.20$\pm$10.755 | 67.00$\pm$13.550 | 7.374 | $<$0.001^*^ |
| **Family adaptability** | 50.88$\pm$10.258 | 52.26$\pm$10.033 **^a^** | 49.36$\pm$10.071 | 47.97$\pm$11.320 | 5.608 | 0.004^*^ |
| **Perceived stress** |  |  |  |  |  |  |
| **Perceived stress scores** | 5.21$\pm$2.536 | 4.02$\pm$2.211 **^a, b^** | 6.35$\pm$1.976 **^b^** | 8.39$\pm$1.661 | 106.753 | $<$0.001^*^ |
| **Perceived stress – Threshold 8** |  | **a, b** | **b** |  | 93.949 | $<$0.001^*^ |
| Normal | 357 (79.7%) | 236 (94.0%) | 109 (67.7%) | 12 (33.3%) |  |  |
| Severe stress | 91 (20.3%) | 15 (6.0%) | 52 (32.3%) | 24 (66.7%) |  |  |
| ^*^ represents statistical significance.  **a** represents statistical difference compared with the moderate psychosomatic distress group, and  **b** represents statistical difference compared with the severe psychosomatic distress group  (after Bonferroni correction for multiple comparisons). | | | | | | |

**Table S22 Multivariate logistic regression analysis of mental health state in female healthcare workers**

| **Variables** |  | | **Model 1** | |  | **Model 2** | |
| --- | --- | --- | --- | --- | --- | --- | --- |
|  |  | | **OR [95% CI]** | ***p* value** |  | **OR [95% CI]** | ***p* value** |
| **Sleep state** | |  | |  |  |  |  |
| Adult attachment style |  | | 1.858 [1.090 3.168] | 0.023 |  | 1.968 [1.164 3.326] | 0.012 |
| Family adaptability |  | | 0.971 [0.948 0.994] | 0.015 |  | 0.968 [0.946 0.991] | 0.006 |
| Perceived stress |  | | 1.321 [1.186 1.472] | $<$0.001 |  | 3.175 [1.886 5.346] | $<$0.001 |
|  |  | |  |  |  |  |  |
| **Emotional state** | |  | |  |  |  |  |
| Educational background |  | | 1.401 [1.038 1.890] | 0.027 |  | 1.521 [1.159 1.995] | 0.002 |
| Utilization of support |  | | - | - |  | 0.871 [0.762 0.995] | 0.042 |
| Family cohesion |  | | 0.970 [0.948 0.993] | 0.011 |  | 0.976 [0.956 0.996] | 0.020 |
| Perceived stress |  | | 2.233 [1.900 2.625] | $<$0.001 |  | 9.649 [5.637 16.519] | $<$0.001 |
|  |  | |  |  |  |  |  |
| **Physical health state** | |  | |  |  |  |  |
| Educational background |  | | 1.601 [1.195 2.146] | 0.002 |  | 1.625 [1.235 2.139] | 0.001 |
| Perceived stress |  | | 1.778 [1.554 2.034] | $<$0.001 |  | 6.359 [3.790 10.669] | $<$0.001 |
| Model 1: Perceived stress score represents perceived stress; Model 2: Perceived stress - threshold 8 represents perceived stress.  OR: Odds ratio; CI: Confidence interval. | | | | | | | |

**Table S23 Multivariate logistic regression analysis of psychosomatic health state in female healthcare workers**

| **Variables** |  | **Model 1** | |  | **Model 2** | |
| --- | --- | --- | --- | --- | --- | --- |
|  |  | **OR [95% CI]** | ***p* value** |  | **OR [95% CI]** | ***p* value** |
| **No psychosomatic distress vs. Moderate psychosomatic distress** | | | | | | |
| Educational background |  | 1.368 [1.043 1.793] | 0.023 |  | 1.452 [1.125 1.875] | 0.004 |
| Family cohesion |  | 0.977 [0.958 0.997] | 0.027 |  | 0.971 [0.953 0.989] | 0.002 |
| Perceived stress |  | 1.688 [1.494 1.907] | $<$0.001 |  | 8.215 [4.359 15.480] | $<$0.001 |
|  |  |  |  |  |  |  |
| **No psychosomatic distress vs. Severe psychosomatic distress** | | | | | | |
| Educational background |  | 2.232 [1.269 3.923] | 0.005 |  | 2.437 [1.421 4.181] | 0.001 |
| Subjective support |  | 0.862 [0.770 0.965] | 0.010 |  | 0.842 [0.760 0.933] | 0.001 |
| Perceived stress |  | 2.938 [2.114 4.084] | $<$0.001 |  | 42.525 [15.214 118.860] | $<$0.001 |
|  |  |  |  |  |  |  |
| **Moderate psychosomatic distress vs. Severe psychosomatic distress** | | | | | | |
| Educational background |  | 1.886 [1.156 3.077] | 0.011 |  | 1.800 [1.137 2.851] | 0.012 |
| Subjective support |  | 0.899 [0.818 0.988] | 0.028 |  | - | - |
| Utilization of support |  | - | - |  | 0.743 [0.587 0.939] | 0.013 |
| Perceived stress |  | 2.061 [1.549 2.743] | $<$0.001 |  | 4.917 [2.185 11.065] | $<$0.001 |
| Model 1: Perceived stress score represents perceived stress; Model 2: Perceived stress - threshold 8 represents perceived stress.  OR: Odds ratio; CI: Confidence interval. | | | | | | |


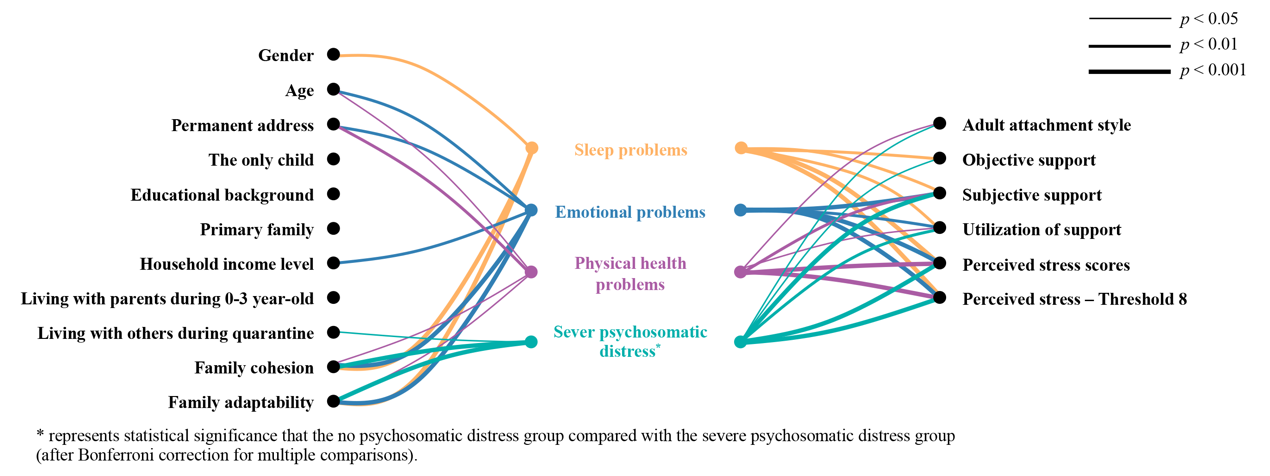


**Figure S1 Comparison of significant factors of mental health-related issues among doctors identified in the univariate analysis**


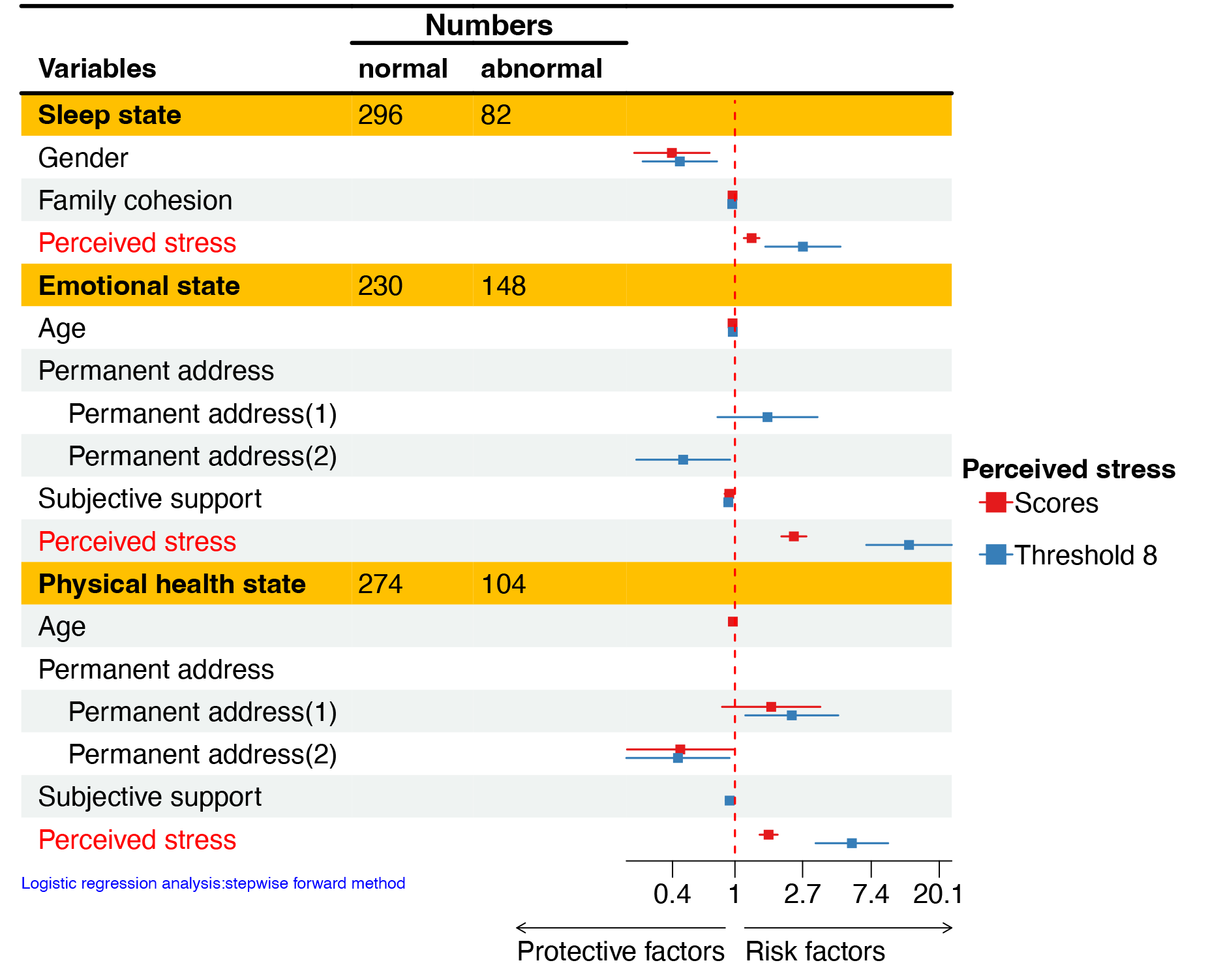


**Figure S2 Odds ratios and 95% Confidence Intervals of the logistic regression analysis of risk and protective factors for mental health problem in doctors**


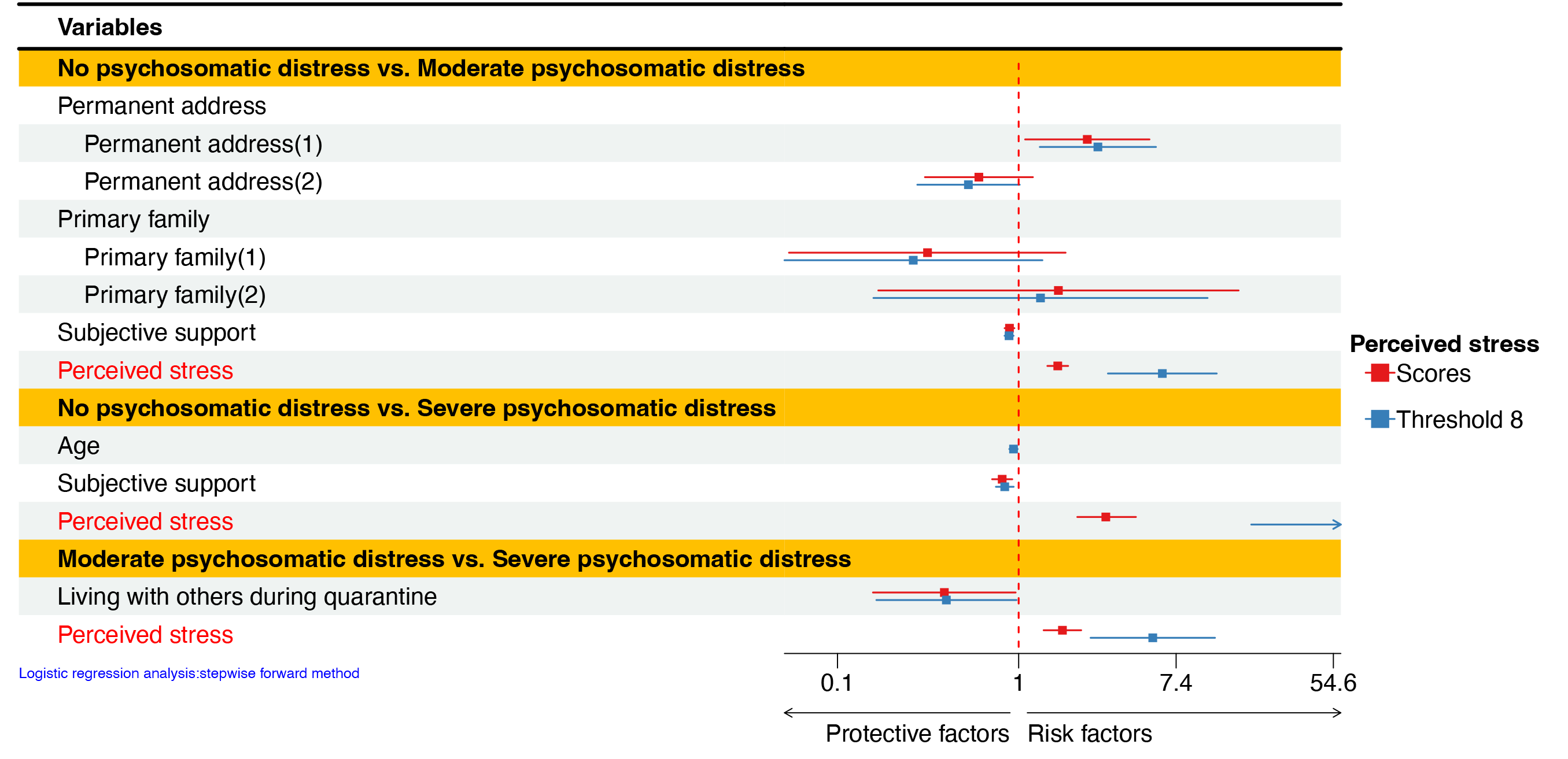


**Figure S3 Odds ratios and 95% Confidence Intervals of the logistic regression analysis of risk and protective factors for psychosomatic distress in doctors**


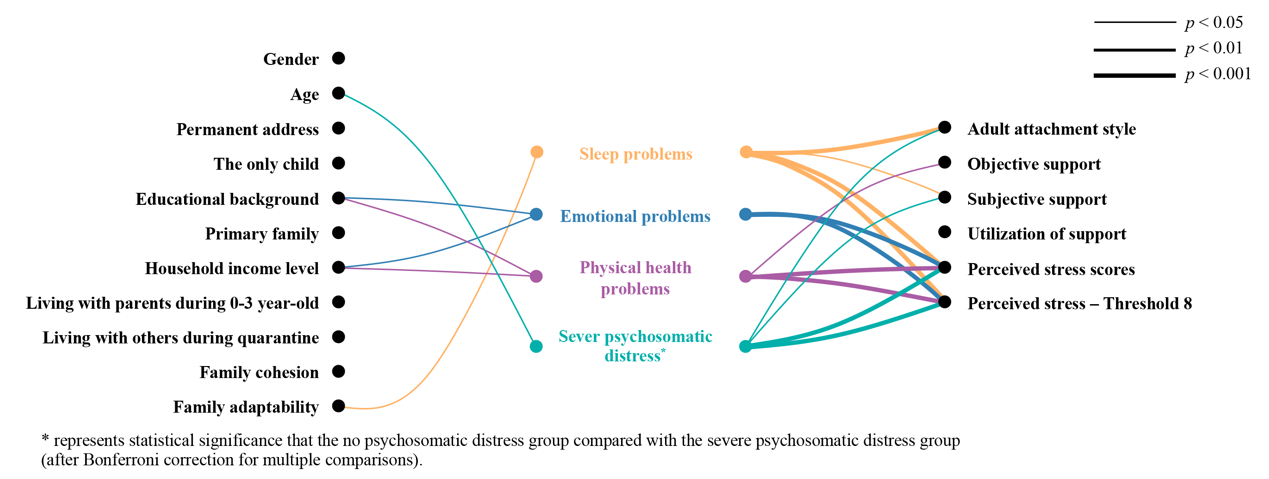


**Figure S4 Comparison of significant factors of mental health-related issues among nurses identified in the univariate analysis**


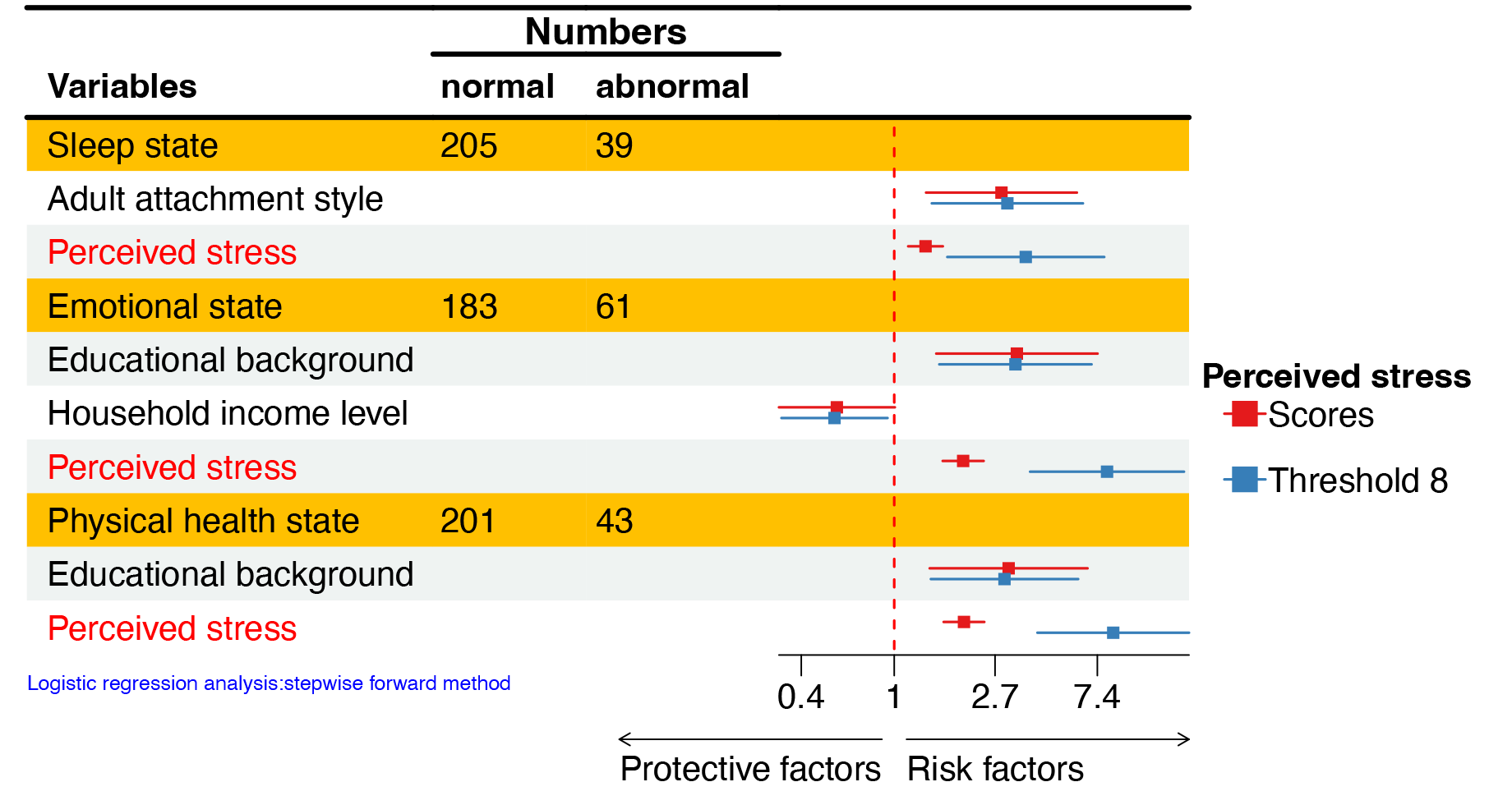


**Figure S5 Odds ratios and 95% Confidence Intervals of the logistic regression analysis of risk and protective factors for mental health problem in nurses**


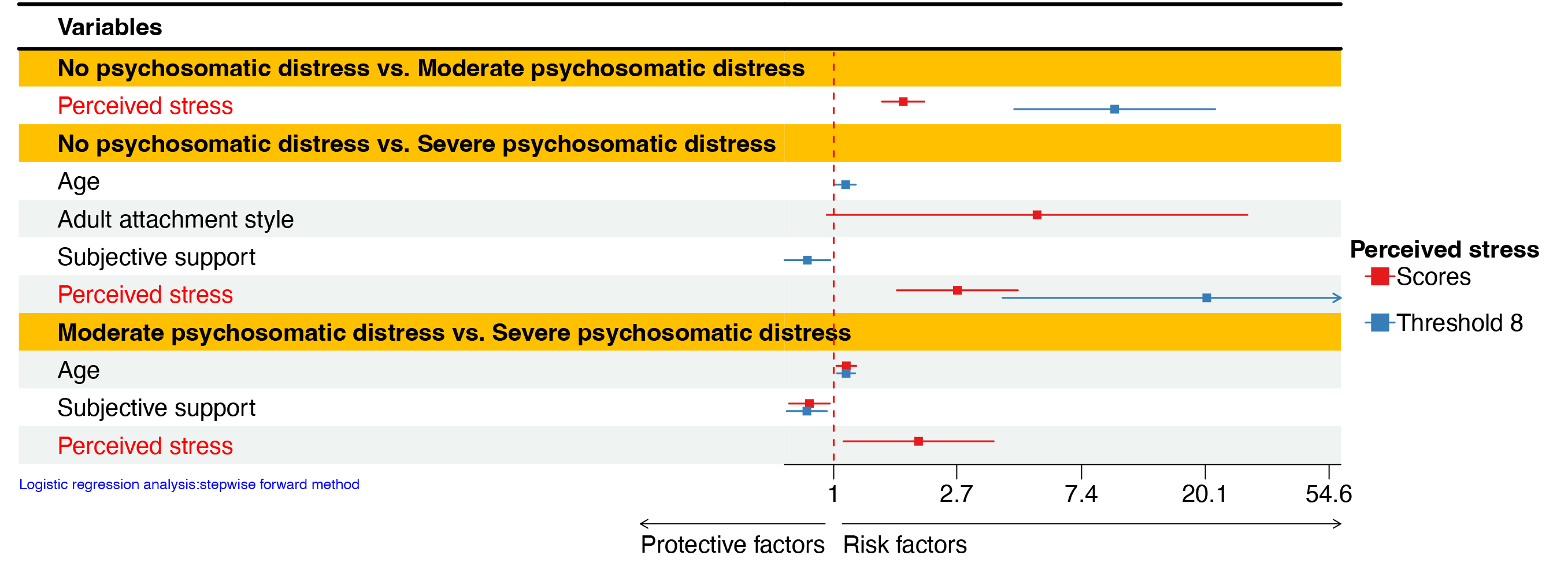


**Figure S6 Odds ratios and 95% Confidence Intervals of the logistic regression analysis of risk and protective factors for psychosomatic distress in nurses**


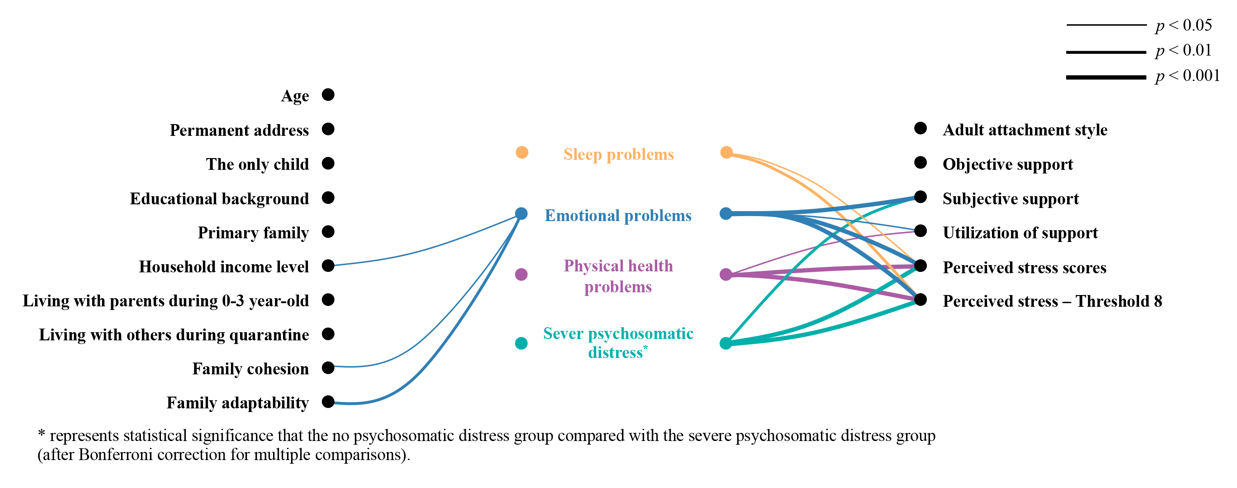


**Figure S7 Comparison of significant factors of mental health-related issues among male doctors and nurses identified in the univariate analysis**


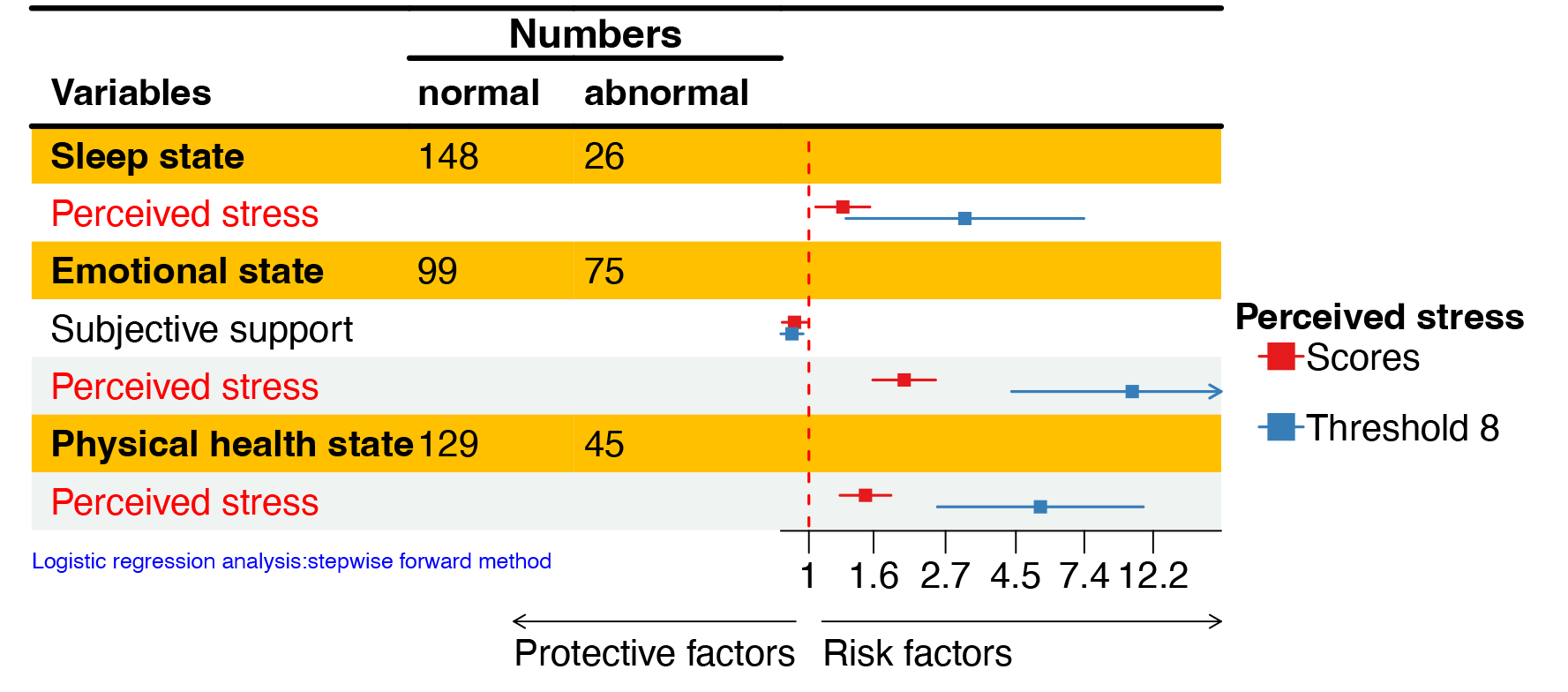


**Figure S8 Odds ratios and 95% Confidence Intervals of the logistic regression analysis of risk and protective factors for mental health problem in male doctors and nurses**


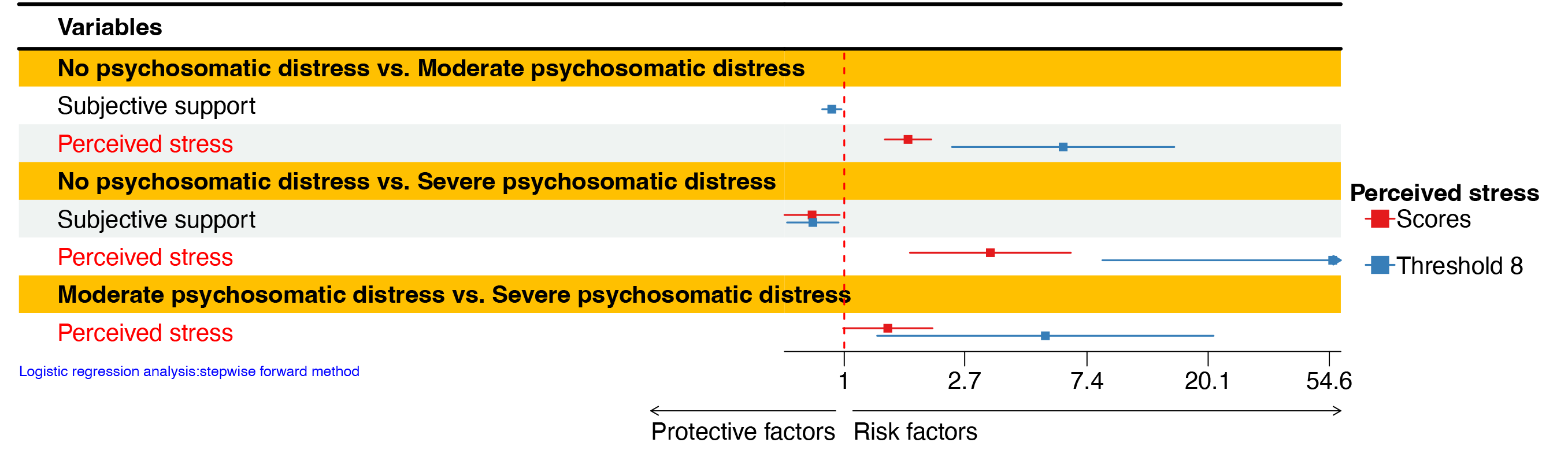


**Figure S9 Odds ratios and 95% Confidence Intervals of the logistic regression analysis of risk and protective factors for psychosomatic distress in male doctors and nurses**


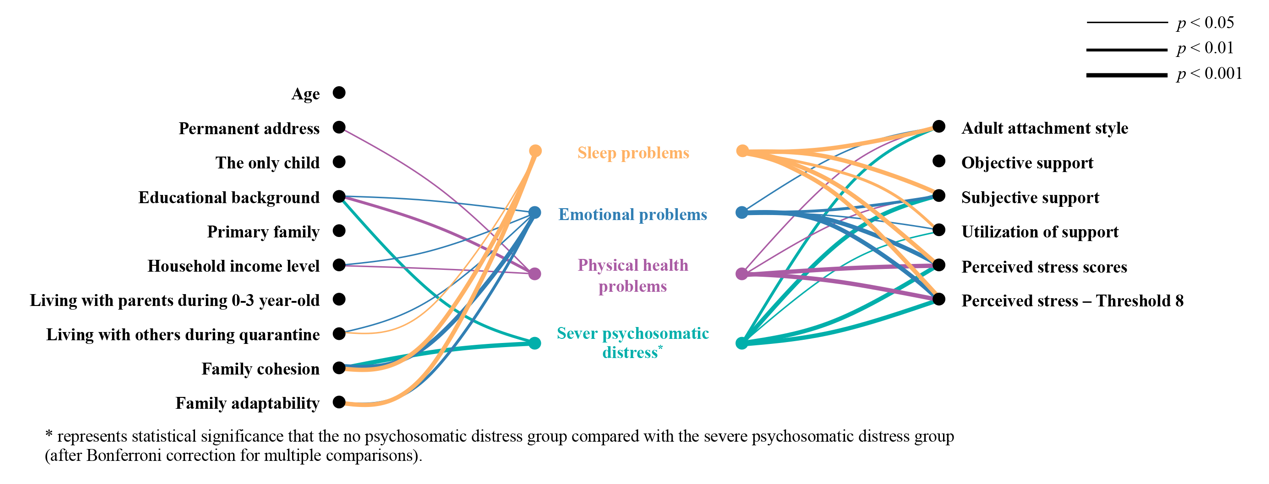


**Figure S10 Comparison of significant factors of mental health-related issues among female doctors and nurses identified in univariate analysis**


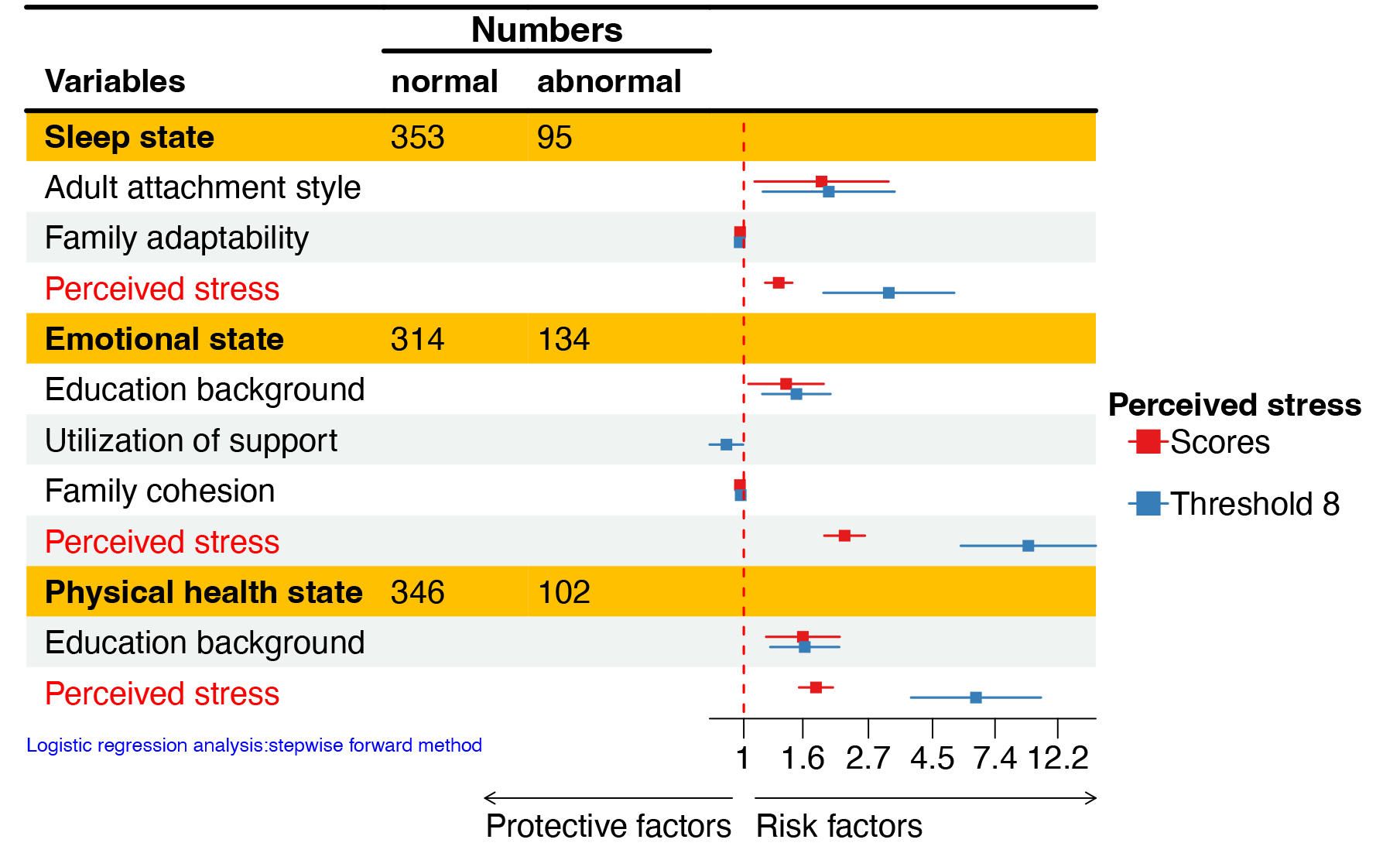


**Figure S11 Odds ratios and 95% Confidence Intervals of the logistic regression analysis of risk and protective factors for mental health problem in female doctors and nurses**


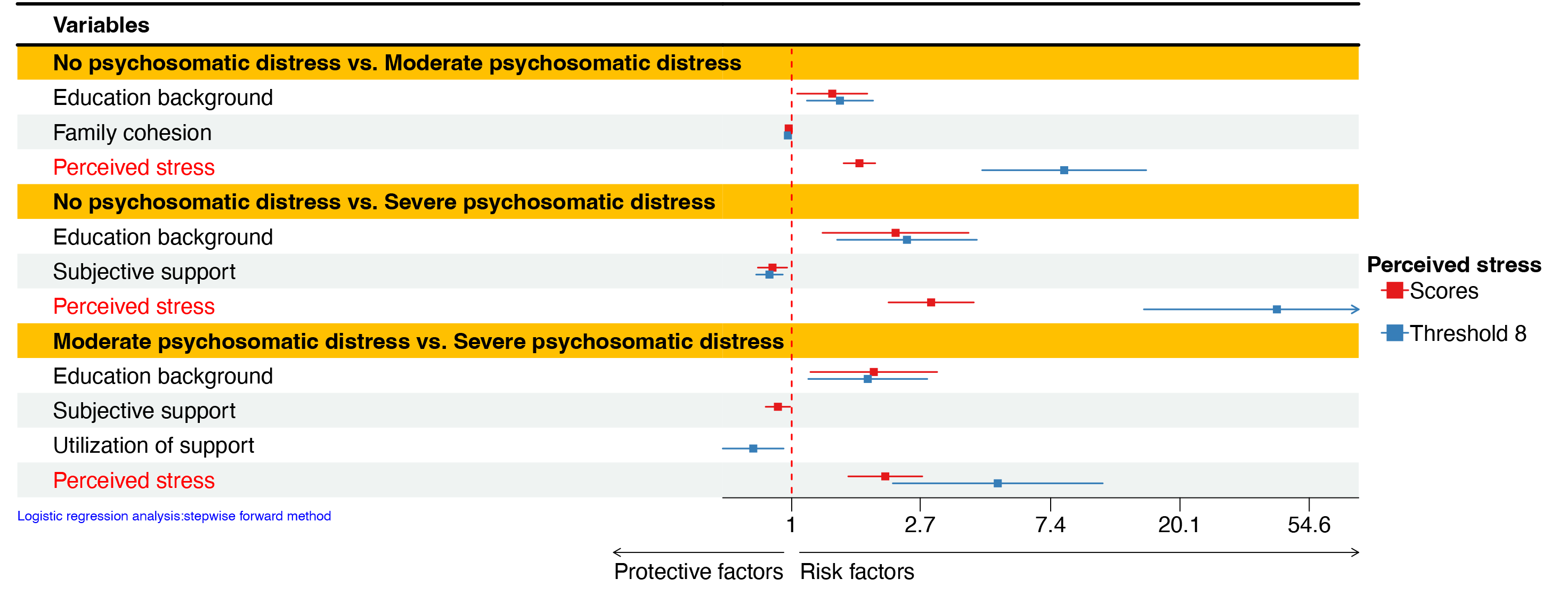


**Figure S12 Odds ratios and 95% Confidence Intervals of the logistic regression analysis of risk and protective factors for psychosomatic distress in female doctors and nurses**
